# Integrating Imaging and Genomics in Amelogenesis Imperfecta: A Novel Diagnostic Approach

**DOI:** 10.3390/genes16070822

**Published:** 2025-07-14

**Authors:** Tina Leban, Aleš Fidler, Katarina Trebušak Podkrajšek, Alenka Pavlič, Tine Tesovnik, Barbara Jenko Bizjan, Blaž Vrhovšek, Robert Šket, Jernej Kovač

**Affiliations:** 1 Department of Paediatric and Preventive Dentistry, Faculty of Medicine, University of Ljubljana, 1000 Ljubljana, Slovenia; alenka.pavlic@mf.uni-lj.si; 2 Department of Endodontics, Faculty of Medicine, University of Ljubljana, 1000 Ljubljana, Slovenia; ales.fidler@mf.uni-lj.si; 3 Department of Endodontics, University Medical Centre Ljubljana, 1000 Ljubljana, Slovenia; 4 Institute of Biochemistry and Molecular Genetics, Faculty of Medicine, University of Ljubljana, 1000 Ljubljana, Slovenia; katarina.trebusakpodkrajsek@mf.uni-lj.si; 5 Clinical Institute of Special Laboratory Diagnostic, University Children’s Hospital, 1000 Ljubljana, Slovenia; tine.tesovnik@kclj.si (T.T.); barbara.jenko.bizjan@kclj.si (B.J.B.); blaz.vrhovsek@kclj.si (B.V.); robert.sket@kclj.si (R.Š.); jernej.kovac@kclj.si (J.K.); 6 Department of Paediatric and Preventive Dentistry, University Medical Centre Ljubljana, 1000 Ljubljana, Slovenia; 7 Faculty of Medicine, University of Ljubljana, 1000 Ljubljana, Slovenia

**Keywords:** amelogenesis imperfecta, radiography, panoramic, molecular genetics, disease-causing variants, imaging genomics

## Abstract

**Background/Objectives:** Amelogenesis imperfecta (AI) represents a heterogeneous group of inherited disorders affecting the quality and quantity of dental enamel, making clinical diagnosis challenging. This study aimed to identify genetic variants in Slovenian patients with non-syndromic AI and to evaluate enamel morphology using radiographic parameters. **Methods:** Whole exome sequencing (WES) was performed on 24 AI patients and their families. Panoramic radiographs (OPTs) were analyzed using Fiji ImageJ to assess crown dimensions, enamel angle (EA), dentine angle (DA), and enamel–dentine mineralization ratio (EDMR) in lower second molar buds, compared to matched controls (*n* = 24). Two observers independently assessed measurements, and non-parametric tests compared EA, DA, and EDMR in patients with and without disease-causing variants (DCVs). Statistical models, including bootstrap-validated random forest and logistic regression, assessed variable influences. **Results:** DCVs were identified in *ENAM* (40% of families), *AMELX* (15%), and *MMP20* (10%), including four novel variants. AI patients showed significant enamel deviations with high reproducibility, particularly in hypomineralized and hypoplastic regions. DA and EDMR showed significant correlations with DCVs (*p* < 0.01). A bootstrap-validated random forest model yielded a 90% (84.0–98.0%) AUC-estimated predictive power. **Conclusions:** These findings highlight a novel and reproducible radiographic approach for detecting developmental enamel defects in AI and support its diagnostic potential.

## 1. Introduction

Enamel defects can be caused by environmental factors, systemic conditions, or inherited genetic variants. In certain conditions, such as dental fluorosis and molar-incisor hypomineralization, both genetic and environmental factors contribute to the defect. Inherited enamel defects are classified as either syndromic, where the genetic variants affect not only the enamel but also other tissues or organs, or non-syndromic, where the enamel is the only tissue affected. Non-syndromic enamel defects fall under the classification of amelogenesis imperfecta (AI) [1].

AI refers to a heterogeneous group of inherited developmental disorders of dental enamel, with an estimated prevalence ranging from 1 in 14,000 to 1 in 700 [2]. Diagnosis of AI is based on clinical and radiographic examination, and/or molecular genetic analysis.

Clinically, AI phenotypes are broadly described as hypoplastic (quantity impairment) and hypomineralized (quality impairment) [3]. The hypoplastic AI shows reduced enamel thickness, such as pitting, partial or generalized missing enamel, and smooth or rough tooth surface due to insufficiently secreted extracellular enamel matrix during amelogenesis [3]. Presumably, mineralization of hypoplastic enamel is rather normal. The hypomineralized AI has enamel of regular thickness but reduced mineralization, and can be further sub-classified as hypomaturation and hypocalcified types. Hypomaturation enamel appears opaque, ranging from white to yellow-brown, with normal thickness but reduced translucency, discoloration, and premature loss [4]. In contrast, hypocalcified enamel is creamy-white to yellow-brown and prone to chipping. Radiographically, hypomature and hypocalcified enamel exhibit radiopacity similar to dentine and lower than dentine, respectively [1]. Some cases may present with both hypoplastic and hypomineralized enamel features, presenting a challenge in classification [5].

To encompass the full spectrum of AI types, various classification schemes have been proposed over the years, based on phenotypes, phenotypes complemented by microradiographic and histopathological findings, phenotypes and modes of inheritance, and finally, phenotypes and inheritance patterns supplemented by insights into causal molecular defects and biochemical outcomes [6]. Despite ongoing calls for a revised AI nosology that incorporates molecular etiology, the traditional clinical and Mendelian inheritance-based classifications remain in use [3].

Since the discovery of the first AI-associated pathogenic variant in the *AMELX* gene [7], significant advances in genetic analysis, including the introduction of next-generation sequencing (NGS), have enabled the identification of an increasing number of causative genes. Thus far, over 100 genes have been identified as being involved in either non-syndromic or syndromic AI, exhibiting various inheritance patterns [8]. Molecular diagnosis enables the identification of causative genes and provides significant insight into the function and pathways of misfolded, improperly translated, or missing proteins. It also indicates individual clustering of phenotypes according to the causative gene, location, and the type of variant [9]. However, variability in expressivity and penetrance patterns contributes to phenotypic diversity and unpredictability [8]. In the sequence variation of the *AMELX* gene located on the X chromosome, AI phenotypes also differ between male and female patients due to lyonization [1].

AI patients may experience hypersensitivity, enamel and dentine loss, gingivitis, and pulp necrosis, depending on the severity of hypomineralization and hypoplasia [10]. Therefore, these patients need timely and appropriate treatment that accounts for the unique characteristics of each disease type; e.g., inadequate enamel mineralization in certain types of AI leads to decreased bonding strength, which consequently reduces the longevity of composite resin restorations [11]. However, clinical examination and genetic analysis alone often do not provide sufficient information for making an unequivocal treatment decision. As radiogenomics emerges as a powerful tool for integrating imaging and genomic data to enhance diagnostics and therapeutics [12], developing additional approaches to assess qualitative and/or quantitative enamel developmental aberrations would be highly beneficial.

The aim of the study was to propose and evaluate the radiographic method for assessing qualitative and/or quantitative aberration of the enamel on panoramic radiographs (OPTs) and evaluate the correlation between the proposed parameters and phenotypes and/or genotypes. Moreover, we sought to pinpoint the genetic etiology of AI without systemic manifestation, hypothesizing that specific diagnostic radiographic features are associated with particular genetic alterations causing AI.

## 2. Materials and Methods

### 2.1. Patients

In the cohort of patients encompassed by this study, we included all AI patients without systemic manifestation, referred to the University Dental Clinic in Ljubljana between 2010 and 2023.

Each AI patient underwent a thorough dental examination, which included an assessment of the pattern of inheritance. For each AI patient, we documented the morphological characteristics of the dentition (e.g., tooth shape, enamel alterations, and color changes), along with any suspected abnormalities of the skin, hair, nails, or bones that could indicate a syndromic condition. Panoramic radiographs (OPTs) were obtained for radiographic evaluation. Further, a blood sample was taken from each AI patient to identify the molecular cause of enamel development deviation.

The clinical diagnosis was made based on the patient’s history, clinical and radiographic data, and genealogical information, all of which were evaluated according to the Witkop and OMIM classification criteria. Before the procedures, we explained the features and objectives of the individual assessments and the aim of the study to each AI patient and their parents. Informed consent for participation was obtained from all subjects involved in the study. The research protocol was approved by the Medical Ethics Committee of the Republic of Slovenia (Act. No.: 0120-505/2020-3).

### 2.2. Molecular Genetic Analysis

Three milliliters of peripheral blood samples were obtained from probands and affected/unaffected participating family members. DNA was isolated using the FlexiGene DNA kit (Qiagene, Hilden, Germany). Whole-exome sequencing (WES) was performed using a DNA prep kit (Illumina, USA) and exome regions were enriched with xGen Exome Hyb Panel v2 probes (IDT, Coralville, Iowa, USA) following the manufacturer’s instructions. Libraries were sequenced with a 150-bp paired-end protocol on an Illumina NovaSeq 6000 sequencing system (Illumina, San Diego, CA, USA). Data were analyzed with the analytical software bcbio-nextgen—v.1.2.9. pipeline (http://doi.org/10.5281/zenodo.3564938; accessed on 14 September 2021), which included Burrows–Wheeler Aligner, gatk4 [13]. Genes were selected based on their involvement in enamel development or associated with the phenotype of enamel developmental abnormalities (according to Human Phenotype Ontology—HPO) (https://hpo.jax.org/app/; accessed on 14 September 2021) [14]. Ontological terms were used: abnormality of dental enamel HP:0000682, amelogenesis imperfecta HP:0000705, dental enamel pits HP:0009722, greyish enamel HP:0000683, hypomature dental enamel HP:0011085, hypomineralization of enamel HP:0006285, hypoplasia of dental enamel HP:0006297 (Table S1). Variant pathogenicity was interpreted according to the American College of Medical Genetics guidelines (ACMG) [15].

### 2.3. Radiographic Analysis

To quantitatively evaluate the altered enamel, we analyzed the OPT images of AI patients and control group patients, who had no developmental enamel disorders and were comparable in age and gender to the AI patients. Image analysis was performed using the open-source platform Fiji ImageJ 2.1.0 [16]. The analysis focused on both lower second permanent molar buds, using the following methods outlined below.

#### 2.3.1. Measurement of Width and Height

We measured two parameters: width (W) and height (H) using the “Straight Lines” tool. For H, a line was drawn vertically from the bottom to the top of the tooth bud, and the distance was recorded. For W, a line was drawn horizontally from the left to the right of the tooth bud, and the distance was measured. The mean values of H and W were calculated for each patient, along with the standard deviation, to assess the variability in measurements.

#### 2.3.2. Measurement of Enamel Angle, Dentine Angle, and Enamel–Dentine Mineralization Ratio

**Figure 1:**
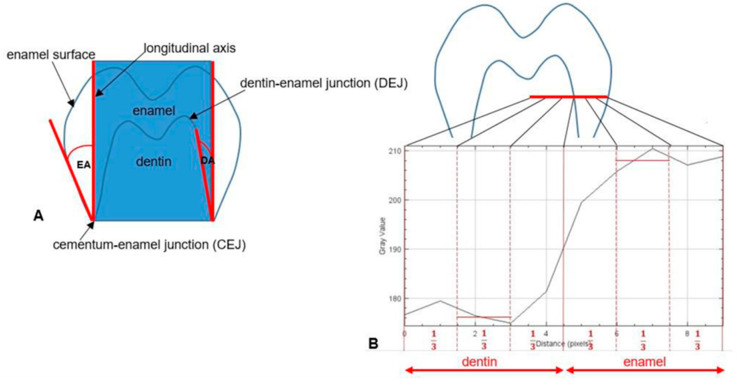
(**A**) The illustration shows the implementation of the enamel angle (EA) and the dentine angle (DA) determined on lower permanent second molar buds, as observed in a panoramic radiograph. (**B**) To assess the relationship between enamel and dentine mineralization, the mean grey values for enamel and dentine (EDMR) are calculated from the middle third of the red-drawn line passing through the enamel and the middle third of the line passing through the dentine, respectively.

We measured three parameters: the enamel angle (EA), dentine angle (DA), and enamel–dentine mineralization ratio (EDMR). To measure EA and DA, we placed an imaginary rectangle on the molar tooth bud (Figure 1A), parallel to the tooth bud’s longitudinal axis, with sides passing through the mesial and distal cementum–enamel junction (CEJ). The EA was measured between the enamel surface and the vertical side of the rectangle, while the DA was measured between the dentine–enamel junction (DEJ) and the vertical side of the rectangle (Figure 1A). These angles were measured using the “Angle” tool in Fiji ImageJ. For each patient, the EA and DA were measured on the mesial and distal sides of both lower second permanent molars, and the mean values with standard deviations were calculated.

The EDMR was calculated as the ratio of enamel to dentine grey values. At half the height of the molar bud crown on the mesial and distal sides, a line was drawn from the dentine towards the enamel surface using the “Straight Lines” tool. The length of the line was twice as long as the enamel thickness. Using the “Plot Profile” tool, we captured the grey values of pixels along the selected line and calculated the mean value from the middle third of the enamel and dentine, respectively (Figure 1B).

For the evaluation of intra-observer and inter-observer reliability, radiographic measurements were independently evaluated by two observers, with one week between re-evaluations.

### 2.4. Statistics Analysis and Model Performance

To assess intra-observer and inter-observer reliability, the intraclass correlation coefficient (ICC) was calculated using IBM SPS [17].

To analyse the dispersion of radiographic parameter results and identify thresholds between AI patients and the control group, the density-based spatial clustering of applications with noise (DBSCAN) method was applied [18].

Subjects were divided into two groups: those with, and those without an identified disease-causing variant (DCV), i.e., a pathogenic or likely pathogenic variant in AI-associated genes. To assess overall differences between these two groups based on radiographic parameters, the non-parametric PERMANOVA test [19] was performed with 9999 permutations and multiple test corrections for false discovery [20]. Individual differences between parameters were assessed using the Mann–Whitney U test [21]. Statistical significance was defined as *p* < 0.05.

Bootstrap-validated random forest [22].and logistic regression [23] models were used to evaluate the impact of individual parameters and predict the presence of DCVs based on these parameters. The models were trained on 70% of randomly selected, balanced data and tested on the remaining 30%. Predictive power and model ranking were assessed using the area under the curve (AUC) of the receiver operating characteristic (ROC) curve. Analyses were conducted using R programming language [24].

## 3. Results

### 3.1. Patients

**Figure 2:**
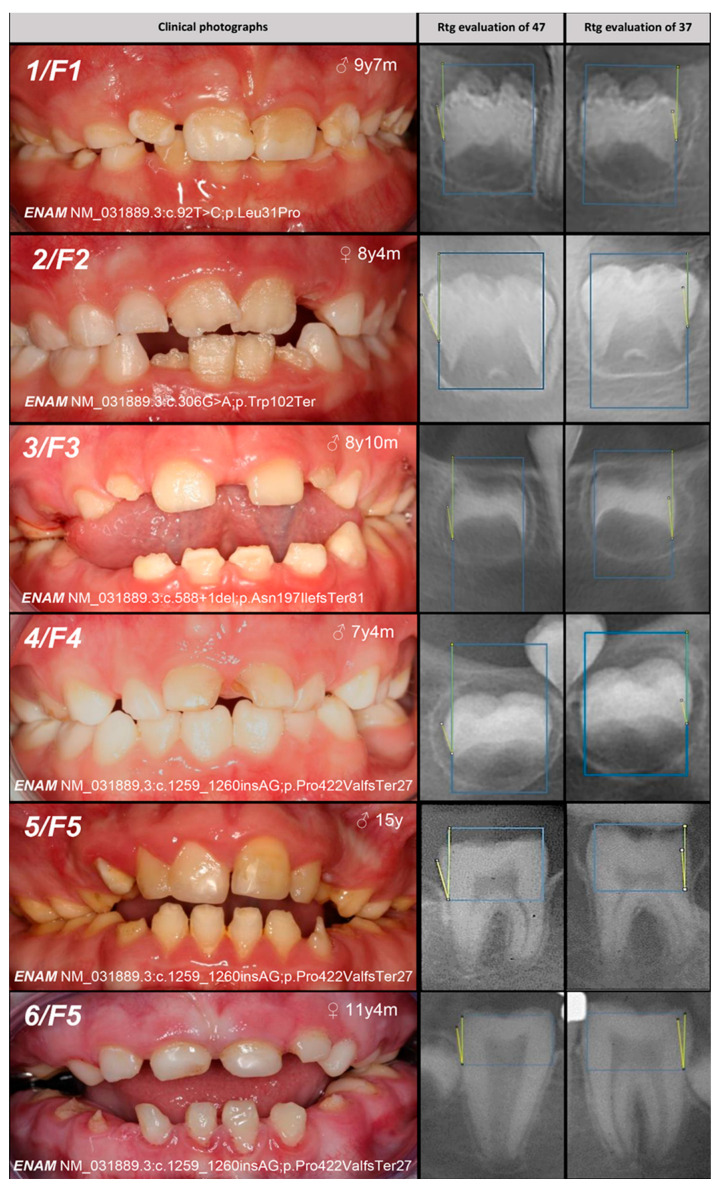

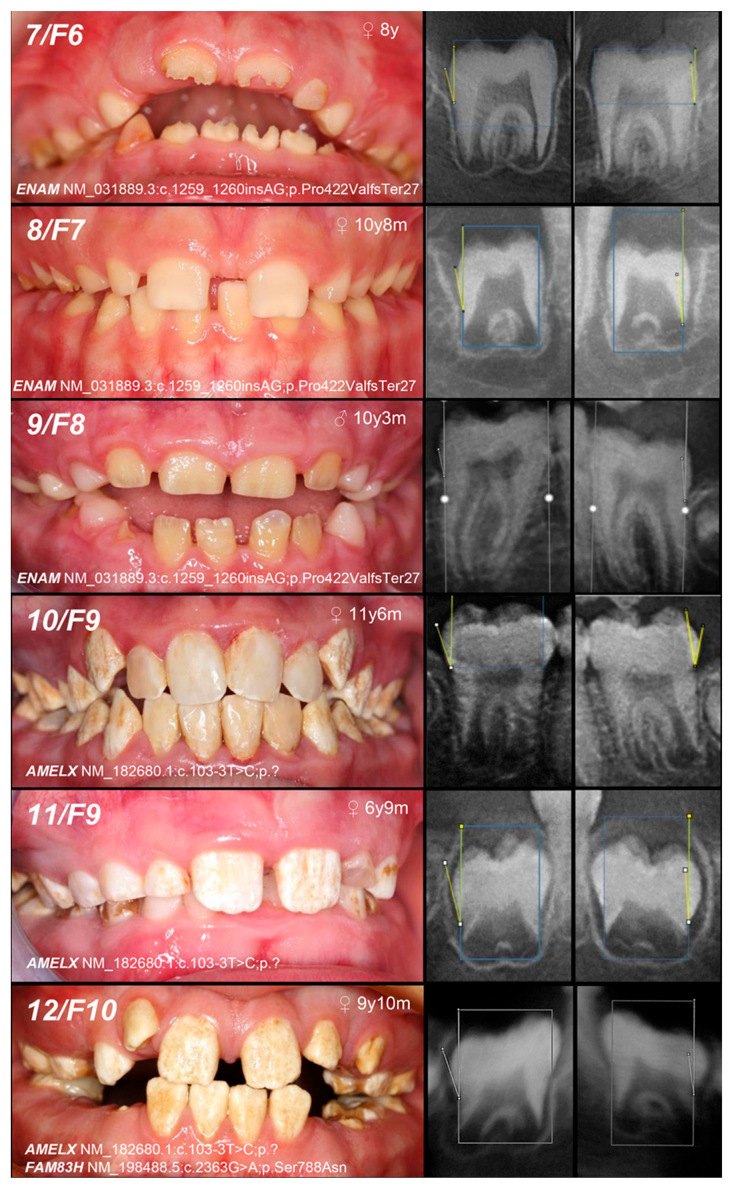

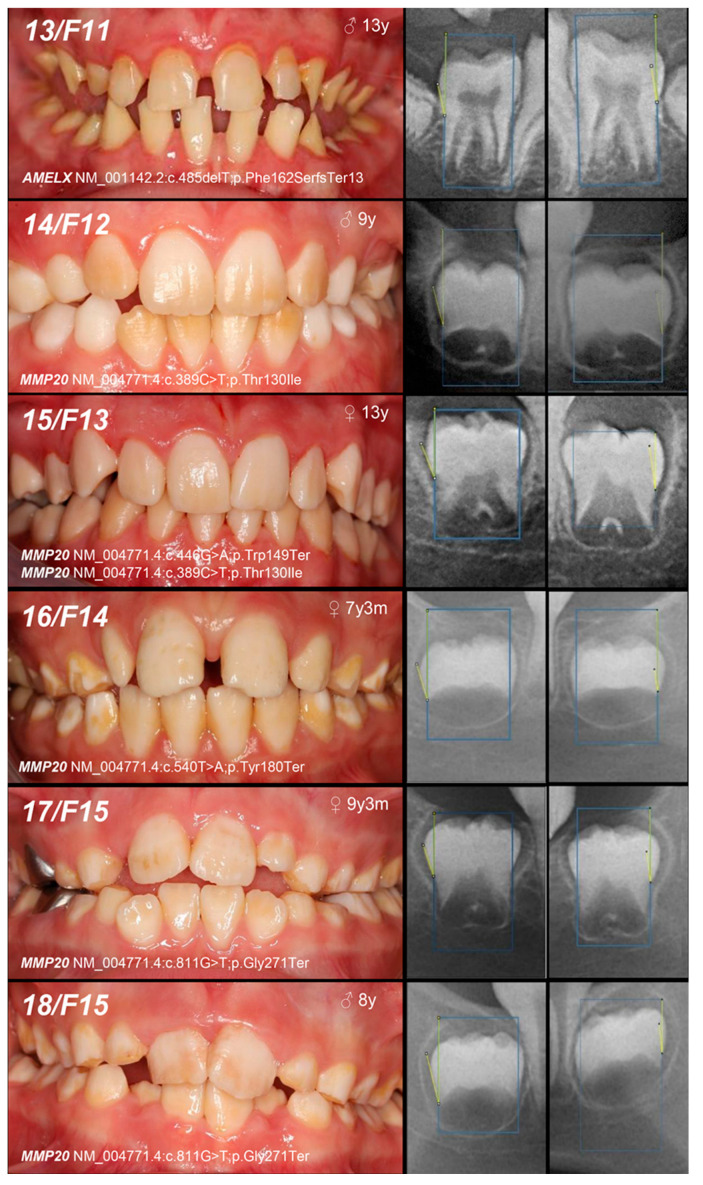

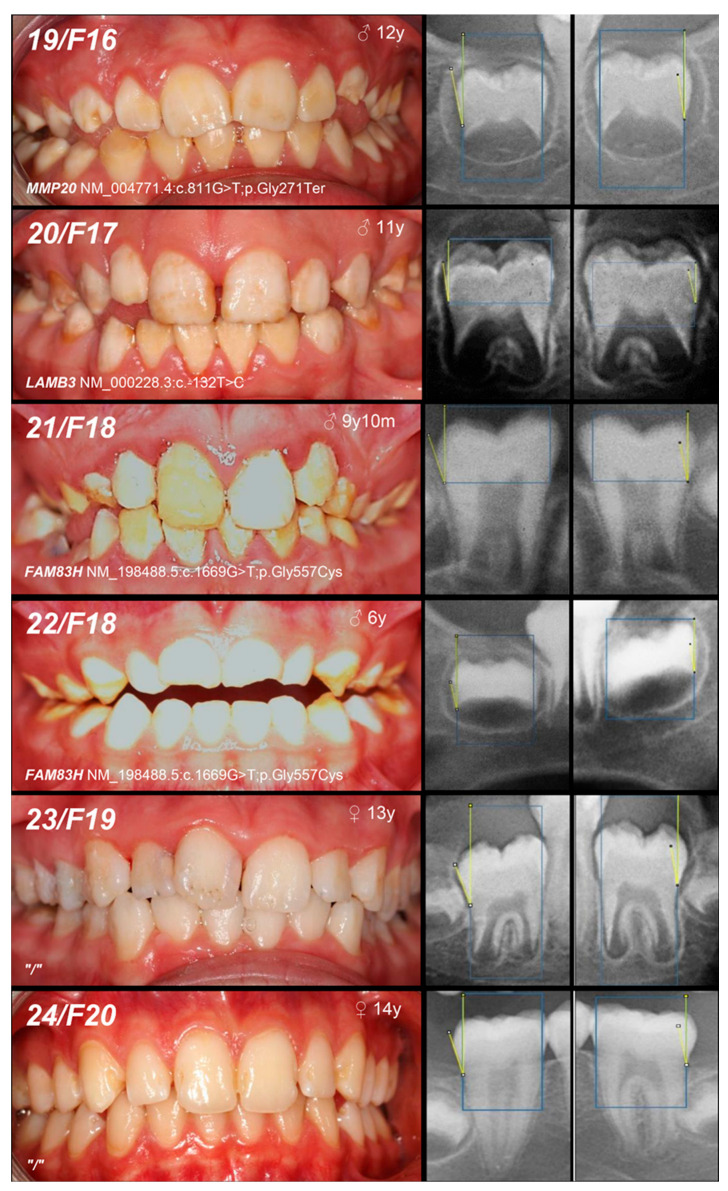
Dental phenotypes (**left**) and radiographic evaluation of tooth buds 37 and 47 (**right**) are shown for each of the 24 AI patients from 20 families. The patient number and family number are shown in the upper left corner of the clinical dental image, while the patient’s age and gender are indicated in the upper right corner. The identified genetic variant is listed in the lower left corner; a slash (“/”) denotes cases in which no convincing genetic variant was identified. The colored lines schematically indicate the measured enamel and dentin angles. Note that the angles were measured on both the mesial and distal sides of each tooth bud.

The study involved 24 Caucasian patients from 20 unrelated families, with the phenotypes of all AI patients illustrated in Figure 2. The inheritance pattern was clinically determined as autosomal dominant (AD) in six families, autosomal recessive (AR) in nine families, and X-linked in three families (Table 1 and Table 2). In two families (F19 and F20), the transmission of enamel development defects could have been either AR or sporadic.

### 3.2. Molecular Analysis

DCVs (pathogenic or likely pathogenic) were identified in 62.5% of AI patients included in the study (Table 1). The remaining 37.5% comprised variants of uncertain significance (VUS), non-conclusive cases, and benign variants (Table 2). In two families (F23 and F24), a definitive molecular diagnosis has not yet been established.

### 3.3. Radiographic Analysis

Intra-observer reliability was high, with intraclass correlation coefficient (ICC) values for W measurements at 0.997, H at 0.995, EA at 0.996, DA at 0.964, and EDMR at 0.983. Inter-observer reliability was good, with ICC values for W at 0.991, H at 0.993, EA at 0.769, DA at 0.913, and EDMR at 0.783.

### 3.4. Statistics Analysis and Model Training

W and H were strongly correlated with each other and their ratios. However, correlations with the identified DCV were not statistically significant (*p* = 0.665). In contrast, the combined correlation of three parameters (EA, DA, and EDMR) with the identified DCV was statistically significant (*p* = 0.0084). Individually, the associations between the identified DCV and DA, as well as EDMR, were statistically significant, with *p*-values of 0.0001189 and 0.005698, respectively. Conversely, the association between the identified DCV and EA was not statistically significant (*p* = 0.2892).

**Figure 3:**
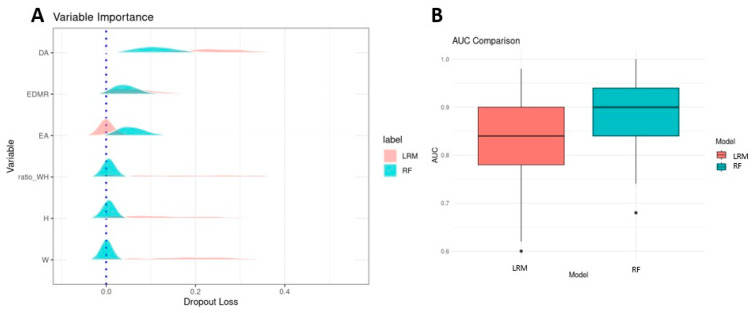
(**A**) Variable importance in predicting the presence of disease-causing variants (DCVs), as determined by two models: random forest (RF, marked in blue) and logistic regression model (LRM, marked in red). The RF model highlights the influence of enamel angle (EA), dentine angle (DA), and the enamel–dentine mineralization ratio (EDMR) on DCV prediction. Width (W), height (H), and their ratio show minimal impact. In contrast, the LRM identifies DA as the most influential predictor, with a moderate contribution from EDMR. The distributions of W, H, and their ratio are long and shallow, likely due to high inter-correlation among these variables. (**B**) Predictive performance of both models across 50 iterations of training and validation, evaluated using the area under the curve (AUC). The RF model (blue) shows a median AUC of 90.0% with an interquartile range (IQR) of 84.0–94.0%, while the LRM (red) shows a median AUC of 84.0% with an IQR of 78.0–90.0%.

Width (W) and height (H) do not significantly enhance the prediction of genetic influence, indicating their minimal contribution to the models. As shown in Figure 3A, the random forest (RF) model demonstrates that W and H have limited impact on the outcome. Including these variables alongside enamel angle (EA), dentine angle (DA), and enamel–dentine mineralization ratio (EDMR) slightly reduced model stability. The RF model incorporating EA, DA, and EDMR achieved a high predictive performance, with an area under the curve (AUC) of 90% (interquartile range: 84.0–94.0%). In contrast, the logistic regression model identified DA and EDMR as the most influential predictors, yielding an AUC of 84% (78.0–90.0%).

**Figure 4:**
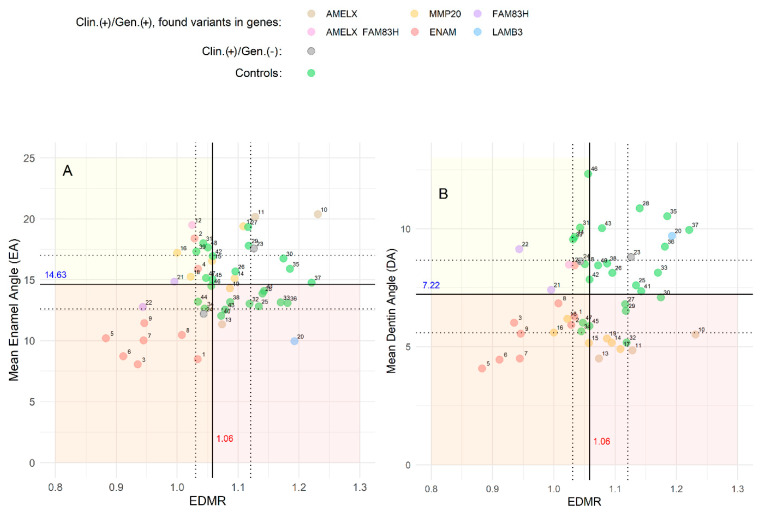
The relationship between (**A**) mean EA and EDMR and (**B**) mean DA and EDMR in AI patients (*n* = 24) and the control group (*n* = 24). Clin.(+)/Gen.(+) refers to affected individuals with identified variants in AI-associated genes. Each dot represents an individual carrying a variant in one of the following genes: red (*ENAM*), brown (*AMELX*), orange (*MMP20*), purple (*FAM83H*), and blue (*LAMB3*). Clin.(+)/Gen.(−) represents affected individuals in whom no convincing pathogenic variant was found in AI-associated genes. Green dots represent unaffected individuals in the control group. The yellow area represents the hypomineralization region, while the red area represents the hypoplastic region. Cut-off values for EA, DA, and EDMR, determined based on the results from healthy teeth in the control group, are 14.63°, 7.22°, and 1.06, respectively.

Based on these findings, we concluded that W, H, and their ratio do not provide significant predictive value for genetic influence, and therefore, EA, DA, and EDMR were used as reference parameters in the graphical analysis presented in Figure 4.

In the AI group (*n* = 24), the results of the mean EA, DA, and EDMR were more dispersed (Figure 4, indicated by colored dots) compared to the control group (*n* = 24) (Figure 4, indicated by green dots). Using the control group data, we established threshold values based on the median with the interquartile range 1.06 for EDMR, and 14.63° and 7.22° for the mean EA and the mean DA, respectively. On both graphs (Figure 4A,B), a yellow-colored area denoted hypomineralization values (EDMR < 1.06), and a reddish-colored area indicated a hypoplastic region (mean EA < 14.63° and mean DA < 7.22° in Figure 4A and Figure 4B, respectively).

Graphical analysis showed that all AI patients with homozygous *ENAM* variants (type IC) and two out of four AI patients with heterozygous *ENAM* variants (type IB) exhibited both hypoplastic and hypomineralized enamel, as indicated by overlapping red and yellow areas in Figure 4A. In patients with AI type IC and three out of four with AI type IB, the graph confirmed hypoplastic and hypomineralized enamel using DA and EDMR (Figure 4B). Interestingly, the remaining patients in type IB only showed enamel hypomineralization.

Enamel analysis of females with *AMELX* variant (type IE) detected hypomineralization in one (Figure 4A; no. 12) and hypoplasia in another (Figure 4B; no. 10 and 11). The hypomineralization of patient no. 12 may relate to an additional variant in the *FAM83H* gene. The only male with *AMELX* variant (no. 13) showed hypoplastic but adequately mineralized enamel.

In both *MMP20* variant patients (IIA2-type), the enamel showed rather normal mineralization, distinctly decreased DA (Figure 4B; no. 14 and 15), while EA values were normal (Figure 4A). In comparison to *MMP20* patients, in both patients carrying *FAM83H* variant (IIIA-type) profound enamel hypomineralization was confirmed (Figure 4A,B; no. 21 and 22).

In the patient with *LAMB3* variant (IA-type), clinically classified as primarily hypoplastic, EA confirmed enamel hypoplasia (red area in Figure 4A; no. 20).

## 4. Discussion

The study results suggest a notable correlation between the proposed radiographic parameters and genetic etiology, emphasizing the potential application of imaging genomics in dentistry. This evaluation could serve as a valuable screening tool for patients and provide deeper insights into the enamel characteristics of individuals with AI, ultimately enabling more personalized and effective dental care.

In the investigation of genetic etiology in 24 cases, four novel DCVs were identified, two in the *ENAM* gene. Clinically expressed as localized rough hypoplastic AI, the missense (NM_031889.3:c.92T>C;p.Leu31Pro) and nonsense (NM_031889.3:c.306G>A;p.Trp102Ter) variants were found in heterozygous form in the male (F1) and female patient (F2), respectively. Milder phenotypes in both heterozygous mothers confirmed variable penetrance (Figures S1 and S2). The *ENAM* frameshift variant (NM_031889.3:c.1259_1260insAG), previously reported [25], was identified in four families in homozygous form associated with AR inheritance and in one family in heterozygous form associated with AD inheritance, suggesting variable expressivity and dose-dependent effect (Figures S4–S8). In the GnomAD database, this variant has a very low population incidence (0.0002517) with no homozygous cases. The frameshift variant (NM_031889.3:c.588+1delG) found in F3 (Figure S3) was also previously reported [25].

In the *AMELX* gene, we identified a novel frameshift variant (NM_182680.1:c.485delT) in a male patient from F11 who had generalized hypoplastic enamel, prominent interdental spacing, and a bilateral posterior open bite. His brother had similarly affected enamel with an anterior open bite, while their mother’s teeth could not be examined as her entire dentition was restored with prosthetic crowns (Figure S11). The patient’s phenotype aligns with reports of individuals with DCVs located near the C-terminal end, i.e., close to this variant’s location [9]. The genotype of three females with an identified DCV in the *AMELX* gene and their clinical presentation of hypomineralized enamel combined with hypoplastic regions was previously reported (Figures S9 and S10) [26].

A novel DCV was also discovered in the *MMP20* gene. In a compound heterozygous female patient from F13, the novel nonsense variant (NM_004771.4:c.446G>A) along with the previously reported missense variant (NM_004771.4:c.389C>T) [27] resulted in hypomineralized enamel with an opaque and creamy appearance (Figure S13). The novel DCV is situated in the zinc-dependent domain, essential for the protein’s catalytic function. Nicolopoulos and colleagues suggested that DCVs in this domain may affect protein stability [4], whereas Kim and colleagues proposed that alterations in the catalytic domain could lead to reduced or complete loss of enzymatic function [28].

The use of a radiography-based tool enabled the quantitative evaluation of the morphological characteristics of developmentally impaired enamel with high reproducibility. To eliminate environmental influences that may contribute to the premature loss of developmentally abnormal enamel or other pathologies (e.g., caries, attrition, abrasion), we evaluated selected parameters on existing OPTs, which were taken in accordance with good clinical practice guidelines in pediatric and preventive dentistry (ADA/FDA Guide to Patient Selection for Dental Radiographs) and the ALARA principle. We assessed lower permanent second molars that had not yet erupted but had fully formed crowns, following Demirjian and colleagues’ criteria for stage D and above [30]. The lower molars were selected for their symmetrical shape, minimal misalignment, rotation, or crowding, and low projection distortion [31].

To the best of our knowledge, this is the first study to establish cut-off values for hypomineralized and hypoplastic enamel. Based on healthy teeth in the control group, we defined cut-off values for EA (14.63°; q1 = 12.5°, q3 = 17°), DA (7.22°; q1 = 5.5°, q3 = 8.75°), and EDMR (1.06; q1 = 1.025, q3 = 1.125). We further identified threshold values for hypomineralized (EDMR < 1.06) and hypoplastic enamel (EA < 14.63° and DA < 7.22°), providing a simple and effective tool to assist dentists in more accurately identifying enamel characteristics.

In cases with DCVs in the *ENAM* gene, radiographic parameters often indicated both hypoplasia and hypomineralization, even though enamel hypomineralization was not clinically diagnosed. The proposed tool confirmed concomitant hypoplastic and hypomineralized enamel in all homozygous patients and in two out of four heterozygous patients. These findings are consistent with observations from scanning electron microscopy studies describing similar enamel abnormalities [5].

In the male patient with the novel DCV in the *AMELX* gene, radiographic parameters confirmed hypoplastic enamel, indicated by lower EA and DA values. Conversely, three female patients from F9 and F10, carrying the same *AMELX* variant, exhibited normal EA, lower DA, and near-normal EDMR values. This aligns with the heterogeneity of *AMELX* phenotypes, influenced by several factors, such as the type and location of DCVs [9], alternative splicing [26], and lyonization in females [1].

Lower DA values observed in *MMP20*-related cases strongly suggest an impact of enamelysin on both DEJ and dentine formation. Indeed, *MMP20* is expressed in odontoblasts and ameloblasts, although its role in dentinogenesis remains unclear. Wang and colleagues reported significantly reduced dentine thickness and mineral density, along with a transient increase in predentine thickness, in an animal model with DCVs in both *MMP20* alleles [32]. Similarly, reduced peritubular dentine thickness was observed in human molars of patients with DCVs in *MMP20* [32]. The authors proposed that improper cleavage of dentine sialoprotein (DSP) due to *MMP20* DCVs leads to deficient peritubular dentine mineralization. The results of this study may indirectly support this theory, as DSP and tuftelin compose the DEJ organic matrix during amelogenesis [33].

Additionally, we confirmed correlations between identified DCVs and DA and EDMR values. These two parameters contribute to predicting DCVs in AI patients, emphasizing the necessity for genetic analysis. With a diagnostic yield of 62.5%, well above the usually reported values in rare disease genetic diagnostics, this study highlights the significant added value of genomics in the clinical algorithms of AI management [34].

However, we acknowledge that due to the low incidence of AI, collaboration with as many institutes as possible worldwide would greatly enhance the quality of data analysis. Given the small sample size in the control group (*n* = 24), the cut-off values for EA (=14.63°), DA (=7.22°), and EDMR (=1.06) should be considered approximate. Future studies should aim to expand the sample size and further investigate the strength of the relationships between these variables. Additionally, the nine genetically unresolved cases out of 24 highlight the need for expanded genomic analysis (e.g., whole genome sequencing), as potential genetic causes of AI may be associated with non-coding regions or yet-to-be-discovered genes.

In conclusion, we propose the use of the described novel radiographic tool to improve the identification of pathological signs of AI. This analysis can be performed on existing OPTs acquired during routine clinical treatment of AI patients, providing a simple yet objective assessment of enamel quality and quantity aberrations. The additional information obtained offers new insight, aiding in more accurate diagnosis and treatment planning for AI.

## Figures and Tables

**Table 1:** Disease-causing variants (DCVs) identified in patients presenting with non-syndromic AI.

Patient (n) Family (n)	Phenotype	OMIM, Mode of Inheritance, Gene Affected	Gene Variant	Zyg.	Protein Outcome	References	Fam. Segreg.	ACMG
1, F1	Hypoplastic (localized)	IB, AD, ***ENAM***	c.92T>C	+/−	p.Leu31Pro	novel	Mo (AC) Fa (U)	likely pathogenic (PP1:moderate, PM5:moderate, PM2:moderate)
2, F2			c.306G>A	+/−	p.Trp102Ter	novel	Mo (AC) FaS (U)	likely pathogenic (PVS1:very strong, PM2:moderate) *
3, F3	Hypoplastic (generalized)		c.588+1del	+/−	p.?	[25]	Mo (U) Fa (AC)	pathogenic (PS4:strong, PVS1:strong, PM2:moderate)
4, F4	Hypomineralized (hypoplastic)	IB, AD, ***ENAM***	c.1259_1260insAG	+/−	p.Pro422Val fsTer27	[5]	Mo (AC) FaS (U)	pathogenic (PS4:strong, PVS1:strong, PM2:moderate) GnomAD: 0.0002517
5, 6, F5	Hypoplastic (generalized—recessive trait; localized, pitting, grooving—dominant trait)	IC, AR/AD, ***ENAM***		−/−		[25]	MoFa (UC)	
7, F6							MoFaS (UC)	
8, F7							MoFa (UC)	
9, F8							MoFa (UC)	
10, 11, F9	Hypomineralized (heterogeneity)	IE, XLD, ***AMELX***	c.103-3T>C	+/−	p.?	[26]	Mo(U) Fa (AC)	likely pathogenic (PP1:strong, PM2:moderate)*
12, F10				+/−			Mo (AC) Fa (NA)	
		and IIIA, AD, ***FAM83H***	c.2363G>A	+/−	p.Ser788Asn	[26]		benign (BA1:stand-alone, BS2:strong, BP4:supporting, BP6:strong)
13, F11	Hypoplastic (heterogeneity)	IE, XLD, ***AMELX***	c.485delT	0/−	p.Phe162SerfsTer13	novel	MoS (A) Fa (NA)	likely pathogenic (PVS1:very strong, PM2:moderate)
14, F12	Hypomineralized (pigmented hypomature)	IIA2, AR, ***MMP20***	c.389C>T	−/−	p.Thr130Ile	[27]	MoFa (UC)	likely pathogenic (PP5: strong, PM2: moderate) GnomAD: 0.001747
15, F13				+/−			Mo (U) Fa (NA)	
			and c.446G>A	+/−	and p.Trp149Ter	novel		likely pathogenic (PVS1:very strong, PM2:moderate) GnomAD: 0.00000398

Inheritance: AD = autosomal dominant, AR = autosomal recessive. Zygosity (Zyg.) is reported in the following format: the symbols (+/−), (−/−), (0/−) denote heterozygous, homozygous, and hemizygous individuals, respectively. Familial segregation (Fam. segreg.) is denoted as follows: Fa (father), Mo (mother), S (sibling), A (affected), U (unaffected), NA (not available), and C (carrier). An asterisk (*) denotes a reclassified variant, confirmed in additional family members or in other families.

**Table 2:** Variants of uncertain significance (VUS), non-conclusive cases, and benign variants identified in patients presenting with non-syndromic AI.

Patient (n) Family (n)	Phenotype	OMIM, Mode of Inheritance, Gene Affected	Gene Variant	Zyg.	Protein Outcome	References	Fam. Segreg.	ACMG
16, F14	Hypomineralized (pigmented hypomature)	IIA2, AR, ***MMP20***	c.540T>A	+/−	p.Tyr180Ter	[28]	Mo (UC) Fa (U)	likely pathogenic (PVS1: very strong, PM2: moderate) GnomAD: 0.00000707
17, 18, F15			c.811G>T	+/−	p.Gly271Ter	novel	Mo (AC) Fa (U)	likely pathogenic (PVS1: very strong, PM2: moderate)
				+/−				
19, F16				+/−			Mo (UC) Fa (U)	
20, F17	Hypoplastic (pitted)	IA, AD, ***LAMB3***	c.-132T>C	+/−	/	novel	Mo (AC) Fa (U)	VUS (PM2: moderate, BP7: supporting)
21, 22, F18	Hypomineralized (hypocalcified)	IIIA, AD, ***FAM83H***	c.1669G>T	+/−	p.Gly557Cys	[29]	MoFa (NA)	benign (BA1: stand-alone, BS1: strong, BS2: supporting, BP4: supporting, BP6: supporting) *
				+/−				

Four variants associated with non-syndromic AI were detected: two are likely pathogenic, one is of uncertain significance (VUS), and one is benign. Variants in families F14, F15, and F16 were non-conclusive, as they were found in only one allele in affected individuals. Inheritance: AD = autosomal dominant, AR = autosomal recessive. Zygosity (Zyg.) is reported in the following format: the symbol (+/−) indicates a heterozygous individual. Familial segregation (Fam. segreg.) is reported as follows: Fa (father), Mo (mother), S (sibling), A (affected), U (unaffected), NA (not available), and C (carrier). The asterisk (*) denotes a variant listed in the ClinVar database with conflicting interpretations: two entries classify the variant as benign, one as VUS, and one as pathogenic.

## Data Availability

The data supporting the findings of this study are available within the article. Raw data are not publicly available due to ethical restrictions and patient confidentiality.

## Supplementary Materials

The following supporting information can be downloaded at: https://www.mdpi.com/article/10.3390/genes16070822/s1, Table S1: List of genes selected based on their involvement in enamel development or in human diseases associated with enamel developmental abnormalities; Figure S1: Family 1: (A) The mixed dentition of a 9-year-and-7-month-old boy (III.2) is characterized by localized, rough hypoplastic AI with superficial exogenous staining. (B) On the panoramic radiograph, a less distinct contrast between enamel and dentine is observed. The posterior dentition exhibits irregular oclusal surfaces. (C) The boy’s mother (II.3) shows minor localized hypoplastic alterations in the posterior region (white arrows) with normal enamel thickness. All her anterior teeth are covered with prosthetics. (D) The family pedigree indicates an autosomal dominant mode of inheritance. Both the boy (III.2) and his mother (II.3) carry a heterozygous *ENAM* variant (NM_031889.3:c.92T>C;p.Leu31Pro). The black arrow indicates the proband, and the asterisk (*) marks the participating family members; Figure S2: Family 2: (A) The mixed dentition of an 8-year-and-4-month-old girl (III.4) shows thin (hypoplastic) enamel on the permanent incisors, which is susceptible to rapid attrition. (B) A panoramic radiograph of the proband reveals abnormal crown formation of the developing permanent incisors due to hypoplastic enamel, with enamel and dentine exhibiting similar radiopacity. (C) The proband’s mother (II.2) presents with an open bite and whitish enamel featuring horizontal grooves (white arrows) in the cervical half of the crowns. (D) The pedigree indicates an autosomal dominant mode of inheritance. Both the girl (III.4) and her mother (II.2) carry a heterozygous *ENAM* variant (NM_031889.3:c.306G>A;p.Trp102Ter). The proband’s father (II.3) shows no signs of enamel abnormalities. The black arrow indicates the proband, the asterisk (*) participating family members, and “NA” non-available family member; Figure S3: Family 3: (A) Clinical photographs of an 8-year-and-10-month-old boy (III.6) show small, smooth, yellow teeth with interdental spacing and an anterior open bite. (B) The panoramic radiograph shows extremely thin enamel, visually absent, covering the entire dentition. (C) The proband’s father (II.8) lacks visible crowns, preventing enamel inspection. (D) The family pedigree is consistent with autosomal dominant AI. Both the boy (III.6) and his father (II.8) carry a heterozygous *ENAM* variant (NM_031889.3:c.588+1del;p.?). The proband’s mother (II.7) is unaffected. The arrow indicates the proband, the asterisk (*) denotes participating family members, and “NA” indicates non-available family members; Figure S4: Family 4: (A) The mixed dentition of a 7-year-and-4-month-old boy (III.7) presents with chalky, whitish enamel featuring localized rough alterations, as well as large mesial carious lesions on both upper deciduous central incisors. (B) The panoramic radiograph shows near-normal morphology, with a lack of contrast between enamel and dentine. (C) The boy’s mother (II.6) presents with whitish enamel and minor localized hypoplastic alterations (white arrows), with normal enamel thickness. His father (II.5) exhibits normally developed dentition. (D) The family pedigree shows that both the boy (III.7) and his mother (II.6) carry a heterozygous *ENAM* variant (NM_031889.3:c.1259_1260insAG;p.Pro422ValfsTer27). The black arrow indicates the proband, and the asterisk (*) denotes participating family members; Figure S5: Family 5: (A) The 15-year-old boy (IV.7) and (B) his 11-year-and-4-month-old sister (IV.8) both exhibit severe, generalized enamel thinning, along with a pronounced anterior open bite. The upper anterior teeth of both patients are restored with composite resin. Additionally, the girl’s permanent first molars are fitted with stainless steel crowns due to significant attrition. (C, D) In both panoramic radiographs, the enamel is poorly visualized. The boy’s upper right permanent canine and upper left permanent central incisor have undergone endodontic treatment. (E) The family pedigree shows that both affected siblings (IV.7 and IV.8) are homozygous for the *ENAM* variant (NM_031889.3:c.1259_1260insAG;p.P422ValfsTer27). (F) Their mother (III.8) and (G) their father (III:7), both of whom have prosthetic crowns, were later confirmed to be heterozygous for the same disease-causing *ENAM* variant; Figure S6: Family 6: (A) The 8-year-old girl (III.1) exhibits extensive hypoplasia of the mixed dentition along with an anterior open bite. The thinness of the enamel is evident on the newly erupted central incisors. (B) No enamel is visible on the panoramic radiograph taken at the age of 9. Neither (C) her mother (II.1) nor (D) her father (II.2) exhibits an aberrant enamel phenotype. (E) In the family pedigree, the girl (III.1) is found to be homozygous for the *ENAM* variant (NM_031889.3:c.1259_1260insAG;p.Pro422ValfsTer27). Both parents (II:1 and II:2), as well as her younger sister (III.2), who was still a baby at the time, were later found to be heterozygous for the same *ENAM* variant. The arrow indicates the proband, the asterisk (*) indicates participating family members, and “NA” denotes non-available family member; Figure S7: Family 7: (A) Clinical photographs of the 10-year-and-8-month-old girl (IV:1) depict severely hypoplastic enamel and interdental spacing. (B) A panoramic radiograph shows mixed dentition with little or no enamel, and noticeably enlarged pulp chambers. (C) The girl’s mother’s (III.4) and (D) father’s teeth (III.3) do not exhibit developmentally aberrant enamel. (E) In the family pedigree, the girl (IV.1) is found to carry homozygous *ENAM* variant (NM_031889.3:c.1259_1260insAG;p.Pro422ValfsTer27). Both parents (III.3 and III.4) were later confirmed to be heterozygous for the same *ENAM* variant. The arrow indicates the proband, and the asterisk (*) indicates participating family members; Figure S8: Family 8: (A) Clinical photographs of the 10-year-and-3-month-old boy (III:5) show generalized thin, hypoplastic enamel and an anterior open bite. (B) On the panoramic radiograph, taken at the age of 12 years, very little enamel is evident—either because it is absent or does not contrast with dentine. (C) His mother’s (II:3) and (D) his father’s teeth (II:4) either have extensive restorations or are covered with crowns or veneers. (E) In the family pedigree, the boy was found to be homozygous for *ENAM* variant (NM_031889.3:c.1259_1260insAG;p.Pro422ValfsTer27). Both parents were heterozygous for the same *ENAM* variant. The arrow indicates the proband, and the asterisk (*) indicates participating family members; Figure S9: Family 9: (A) Clinical photographs of the 11-year-and-6-month-old girl (IV.4) and (B) her 6-year-and-9-month-old sister (IV.5) show similarly altered enamel, with a chalky-white to yellowish color featuring hypoplastic defects and marked hypomineralization. Both panoramic radiographs, (C) taken at the age of 9 years and (D) 6 years and 9 months, respectively, show enamel of adequate thickness but with radiopacity more similar to dentine. (E) The family pedigree indicates an X-linked mode of inheritance. Both sisters (IV.4 and IV.5) were identified as heterozygous for an *AMELX* variant (NM_182680.1:c.103-3T>C;p.?). (F) The probands’ father (III.6), who was later found to be hemizygous for the same *AMELX* variant, shows teeth with developmentally aberrant enamel. The mother’s teeth (III.7) developed normally. The arrows indicate probands, the asterisk (*) indicates participating family members, and “NA” denotes non-available family members; Figure S10: Family 10: (A) The hypomineralized mixed dentition of the 9-year-and-10-month-old girl (III.6) reveals chalky-white to yellowish hypoplastic enamel on all deciduous and permanent teeth. Profound attrition is observed on the occlusal surfaces of posterior teeth, with extensive glass ionomer fillings covering the permanent first molars. (B) The panoramic radiograph shows enamel of normal thickness but lacking contrast between enamel and dentine. (C) The family pedigree indicates an X-linked mode of inheritance: the girl (III.6) and her mother (II.4) are heterozygous for the *AMELX* variant (NM_182680.1:c.103-3T>C;p.?) and an additional *FAM83H* variant (NM_198488.3:c.2363G>A;p.Ser788Asn). (D) The proband’s mother (II.4) had no crowns available for inspection. Her upper jaw was toothless, and only canine teeth and a removable partial denture were present in the lower jaw; Figure S11: Family 11: (A) The permanent dentition of the 13-year-old boy (III:14) shows generalized thin, hypoplastic enamel with prominent interdental spacing. (B) Very little enamel is evident on the panoramic radiograph. The first permanent molars appear taurodontic. (C) The boy’s brother’s teeth (III.13) also show developmentally aberrant enamel, an open bite, and marginal gingivitis. (D) The family pedigree indicates an X-linked mode of inheritance: the boy (III.14) and his older brother (III.13) carry an *AMELX* variant (NM_001142.2:c.485delT;p.Phe162SerfsTer13) in hemizygous form, while their mother (II.8) carries the same *AMELX* variant in heterozygous form. (E) The boy’s mother’s teeth (II.8) lack crowns for inspection. A dental examination of the father (II.7) was not possible. The arrow indicates the proband, and the asterisk (*) denotes participating family members; Figure S12: Family 12: (A) The mixed dentition of the 9-year-old boy (III.1) shows a generalized yellowish dull discoloration of the enamel, especially in the permanent teeth. (B) The panoramic radiograph reveals reduced contrast between enamel and dentine. In both parents, (C) his mother’s teeth (II.2) and (D) his father’s teeth (II.3), no aberrant enamel phenotype is detected. (E) The family pedigree shows that the boy (III.1) carries a homozygous *MMP20* variant (NM_004771.4:c.389C>T;p.Thr130Ile). Both parents (II.2 and II.3) were later found to be heterozygous for the same *MMP20* variant. The arrow indicates the proband, and the asterisk (*) denotes participating family members; Figure S13: Family 13: (A) The enamel of the 13-year-old girl (III.2) shows an opaque and creamy appearance. (B) A panoramic radiograph taken at the age of 12 years reveals normal morphology of tooth germs, with limited contrast between enamel and dentine. (C) The family pedigree shows that the patient carries compound heterozygous *MMP20* variants [NM_004771.4:c.446G>A;p.Trp149Ter and NM_004771.4:c.389C>T;p.Thr130Ile]. Her father (II.6) passed away, and DNA from her unaffected mother (II.5) was not available. The arrow indicates the proband, and asterisk (*) denotes participating family member.
