# Supplementary material for: Integrating Imaging and Genomics in Amelogenesis Imperfecta: A Novel Diagnostic Approach
Source: Genes (Basel). 2025 Jul 14;16(7):822. doi: 10.3390/genes16070822 (PMC12294184; doi:10.3390/genes16070822)
Supplement: Supplementary file 1 [file genes-16-00822-s001.zip › genes-3733342-SM.pdf]

Supplementary Materials

Table S1: List of genes selected based on their involvement in enamel development or in human diseases associated with enamel developmental abnormalities.

AARS1	*ACP4*	*ADGRV1*	*AIRE*	*AKT1*	*ALDH3A2*	*AMBN*	*AMELX*
AMTN	*ANAPC1*	*APC*	*ARVCF*	*ATR*	*ATRIP*	*AXIN2*	*BAZ1B*
BCL7B	*BUD23*	*CARS1*	*CCDC8*	*CDH23*	*CENPE*	*CENPJ*	*CEP152*
CHD3	*CIB2*	*CLDN1*	*CLDN19*	*CLEC7A*	*CLIP2*	*CNNM4*	*COG6*
COL17A1	*COL7A1*	*COMT*	*COX7B*	*CREBBP*	*CRTAP*	*CTBP1*	*CTSK*
CUL7	*CYP27B1*	*CYP2R1*	*DDX59*	*DHCR7*	*DIAPH1*	*DLX3*	*DNA2*
DNAJC21	*DNAJC30*	*DSPP*	*EDA*	*EDARADD*	*EIF4H*	*ELMO2*	*ELN*
ENAM	*EP300*	*ERCC1*	*ERCC2*	*ERCC3*	*ERCC4*	*ERCC6*	*ERCC8*
ESPN	*FAM20A*	*FAM20C*	*FAM83H*	*FBXO28*	*FERMT1*	*FGF10*	*FGF3*
FGFR1	*FGFR2*	*FGFR3*	*FKBP6*	*FLNB*	*GALNS*	*GALNT3*	*GJA1*
GLB1	*GNAS*	*GNB2*	*GP1BB*	*GPR68*	*GRHL2*	*GTF2E2*	*GTF2H5*
GTF2I	*GTF2IRD1*	*GTF2IRD2*	*HCCS*	*HIRA*	*HLA-DQA1*	*HLA-DQB1*	*HRAS*
IFT122	*IFT43*	*IFT52*	*IKBKG*	*IL17F*	*IL17RA*	*IL17RC*	*IRF6*
IRX5	*ITGA6*	*ITGB4*	*ITGB6*	*JMJD1C*	*KCNJ2*	*KLK4*	*KRT14*
KRT5	*LAMA3*	*LAMB3*	*LAMC2*	*LIMK1*	*LMX1B*	*LONP1*	*LRP4*
LRP6	*LTBP3*	*MBTPS2*	*METTL27*	*MLXIPL*	*MMP1*	*MMP20*	*MPLKIP*
MSX1	*MYO7A*	*NCF1*	*NDUFB11*	*NECTIN1*	*NECTIN4*	*NF1*	*NUP133*
NUP85	*OBSL1*	*OCRL*	*ODAPH*	*OFD1*	*ORAI1*	*P4HB*	*PAK2*
PAX9	*PCDH15*	*PCNT*	*PDE4D*	*PDZD7*	*PEX1*	*PEX6*	*PGAP1*
PHEX	*PIK3C2A*	*PIK3R1*	*PLEC*	*PLK4*	*POLR1B*	*POLR1C*	*POLR1D*
PORCN	*PTDSS1*	*PTEN*	*RAD21*	*RBBP8*	*RECQL4*	*RELT*	*RFC2*
RHOA	*RNF113A*	*RNU12*	*ROGDI*	*RREB1*	*RUNX2*	*SATB1*	*SCUBE3*
SEC24C	*SEC24D*	*SLC10A7*	*SLC13A5*	*SLC24A4*	*SLC29A3*	*SLC35A2*	*SMARCA2*
SMARCD2	*SMOC2*	*SP6*	*STIM1*	*STX16*	*STX1A*	*SUMO1*	*TARS1*
TBCE	*TBL2*	*TBX1*	*TCIRG1*	*TCOF1*	*TGFA*	*TMEM165*	*TMEM270*
TNFRSF11A	*TP63*	*TRAF3IP2*	*TRAIP*	*TRIM37*	*TSC1*	*TTC7A*	*UFD1*
USH1C	*USH1G*	*USH2A*	*VDR*	*VPS37D*	*WDR19*	*WDR35*	*WDR72*
WHRN	*WNT10A*	*WNT10B*					

FAMILY 1

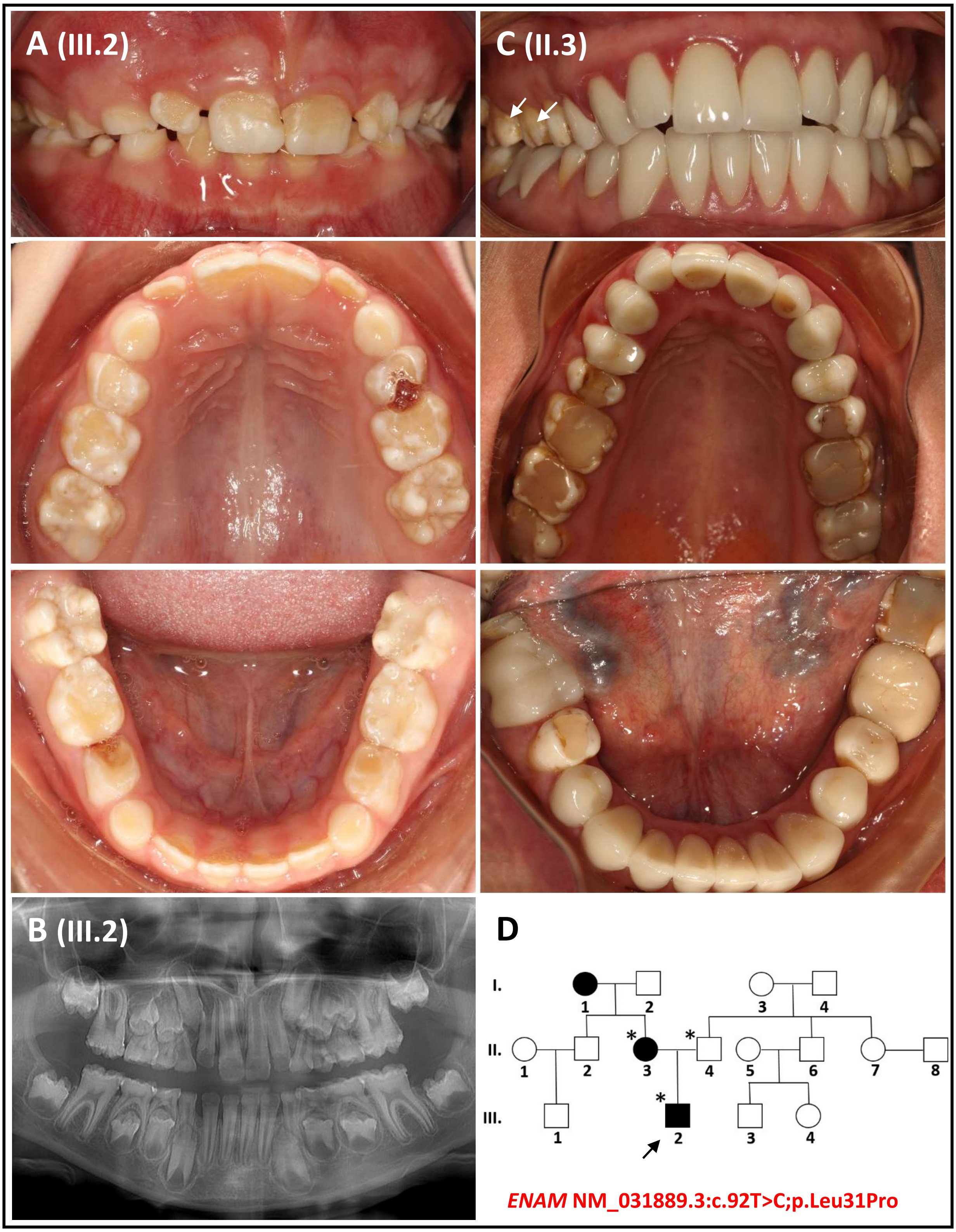

Figure S1. Family 1: **(A)** The mixed dentition of a 9-year-and-7-month-old boy (III.2) is characterized by localized, rough hypoplastic AI with superficial exogenous staining. **(B)** On the panoramic radiograph, a less distinct contrast between enamel and dentine is observed. The posterior dentition exhibits irregular oclusal surfaces. **(C)** The boy's mother (II.3) shows minor localized hypoplastic alterations in the posterior region (white arrows) with normal enamel thickness. All her anterior teeth are covered with prosthetics. **(D)** The family pedigree indicates an autosomal dominant mode of inheritance. Both the boy (III.2) and his mother (II.3) carry a heterozygous *ENAM* variant (NM_031889.3:c.92T>C;p.Leu31Pro). The black arrow indicates the proband, and the asterisk (*) marks the participating family members.

FAMILY 2

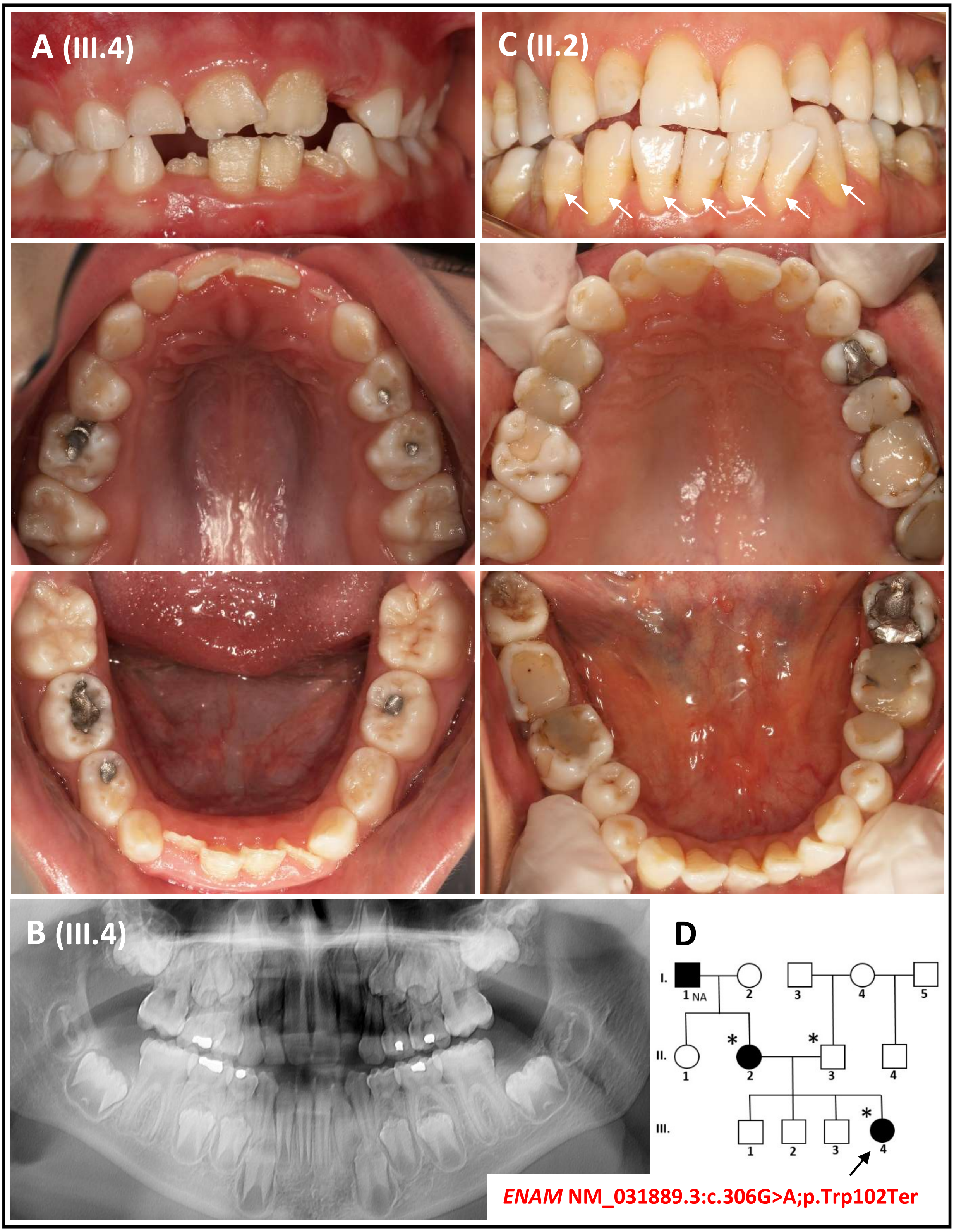

Figure S2. Family 2: **(A)** The mixed dentition of an 8-year-and-4-month-old girl (III.4) shows thin (hypoplastic) enamel on the permanent incisors, which is susceptible to rapid attrition. **(B)** A panoramic radiograph of the proband reveals abnormal crown formation of the developing permanent incisors due to hypoplastic enamel, with enamel and dentine exhibiting similar radiopacity. **(C)** The proband's mother (II.2) presents with an open bite and whitish enamel featuring horizontal grooves (white arrows) in the cervical half of the crowns. **(D)** The pedigree indicates an autosomal dominant mode of inheritance. Both the girl (III.4) and her mother (II.2) carry a heterozygous *ENAM* variant (NM_031889.3:c.306G>A;p.Trp102Ter). The proband's father (II.3) shows no signs of enamel abnormalities. The black arrow indicates the proband, the asterisk (*) participating family members, and "NA" non-available family member.

FAMILY 3

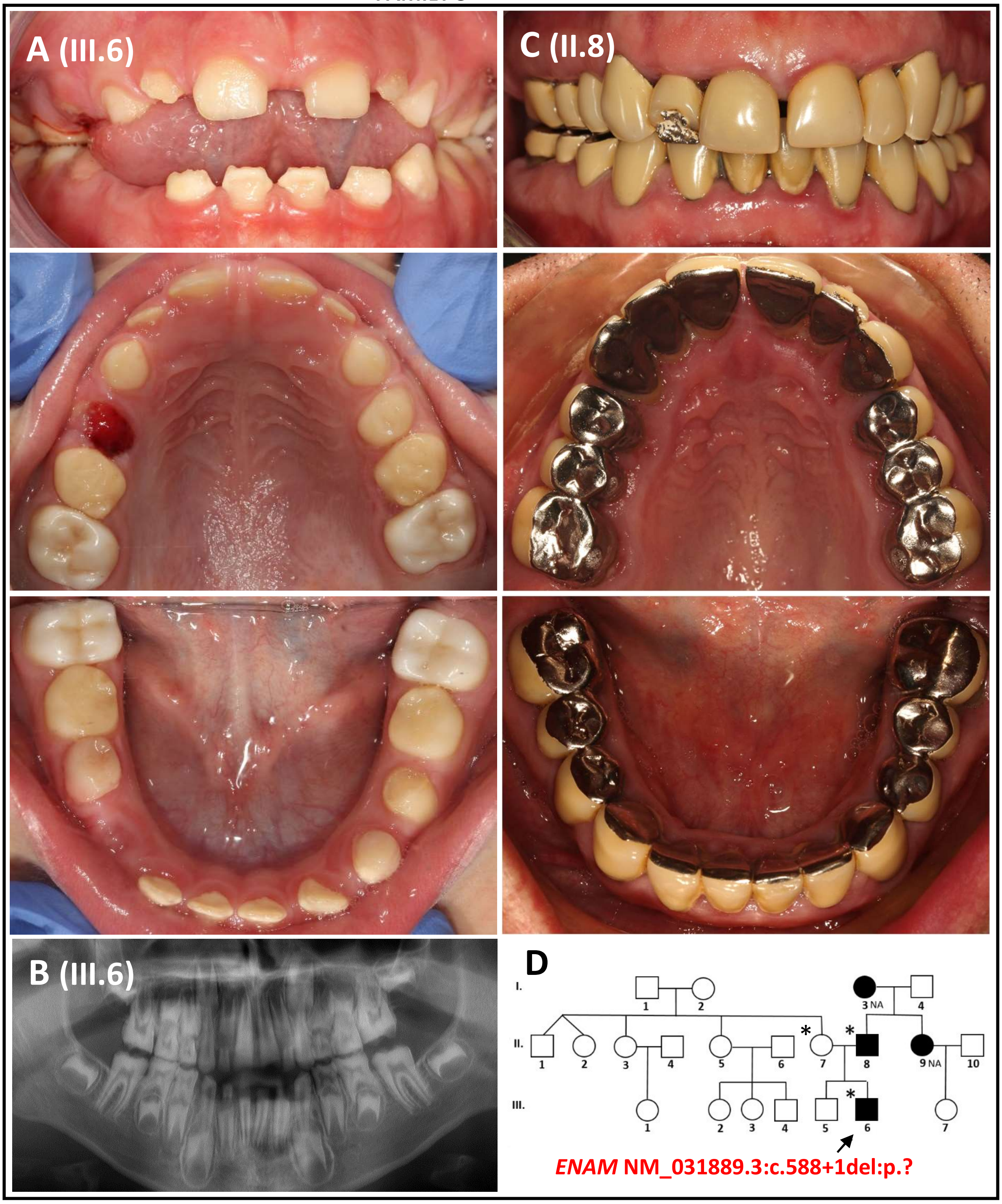

Figure S3. Family 3: **(A)** Clinical photographs of an 8-year-and-10-month-old boy (III.6) show small, smooth, yellow teeth with interdental spacing and an anterior open bite. **(B)** The panoramic radiograph shows extremely thin enamel, visually absent, covering the entire dentition. **(C)** The proband's father (II.8) lacks visible crowns, preventing enamel inspection. **(D)** The family pedigree is consistent with autosomal dominant AI. Both the boy (III.6) and his father (II.8) carry a heterozygous *ENAM* variant (NM_031889.3:c.588+1del;p.?). The proband's mother (II.7) is unaffected. The arrow indicates the proband, the asterisk (*) denotes participating family members, and "NA" indicates non-available family members.

FAMILY 4

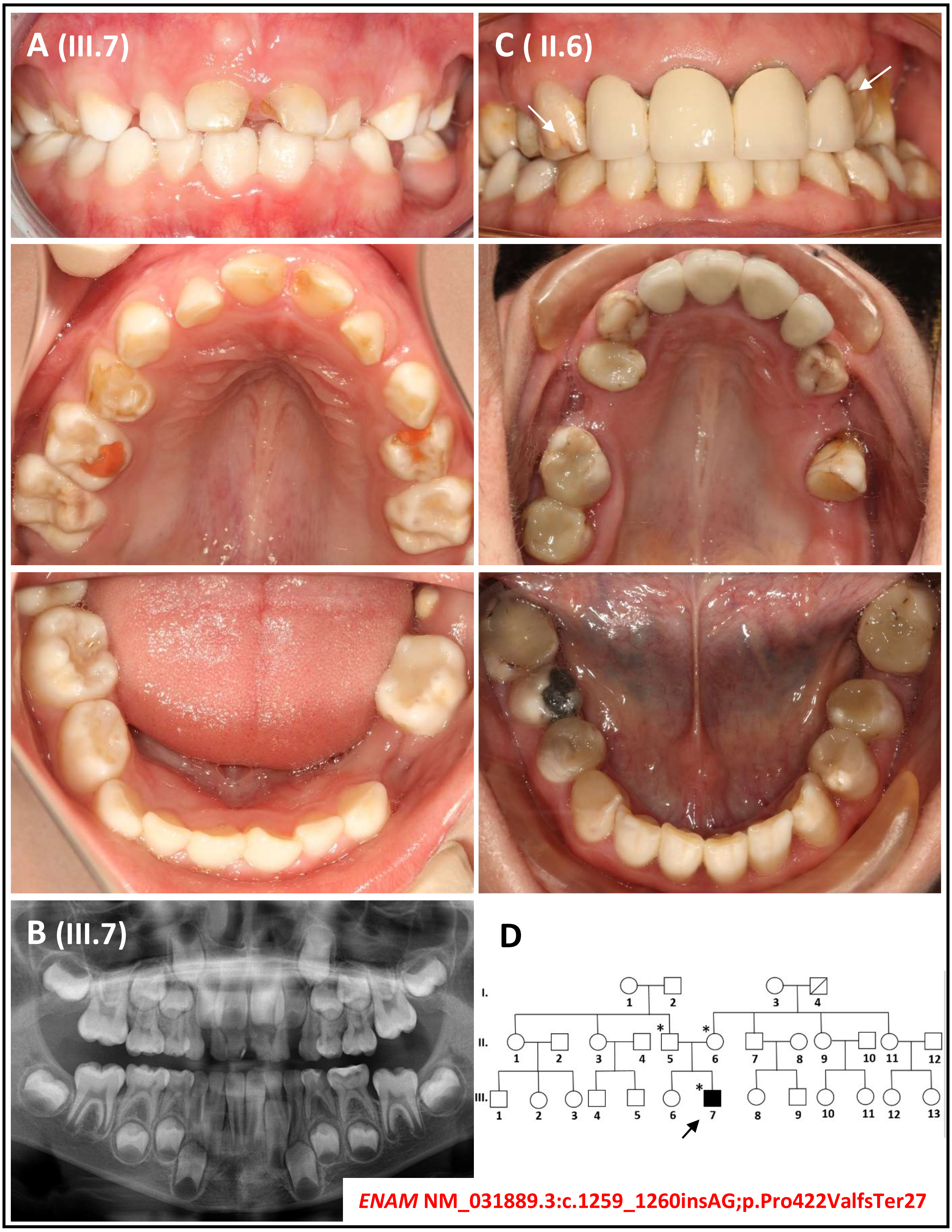

Figure S4. Family 4: **(A)** The mixed dentition of a 7-year-and-4-month-old boy (III.7) presents with chalky, whitish enamel featuring localized rough alterations, as well as large mesial carious lesions on both upper deciduous central incisors. **(B)** The panoramic radiograph shows near-normal morphology, with a lack of contrast between enamel and dentine. **(C)** The boy's mother (II.6) presents with whitish enamel and minor localized hypoplastic alterations (white arrows), with normal enamel thickness. His father (II.5) exhibits normally developed dentition. **(D)** The family pedigree shows that both the boy (III.7) and his mother (II.6) carry a heterozygous *ENAM* variant (NM_031889.3:c.1259_1260insAG;p.Pro422ValfsTer27). The black arrow indicates the proband, and the asterisk (*) denotes participating family members.

FAMILY 5

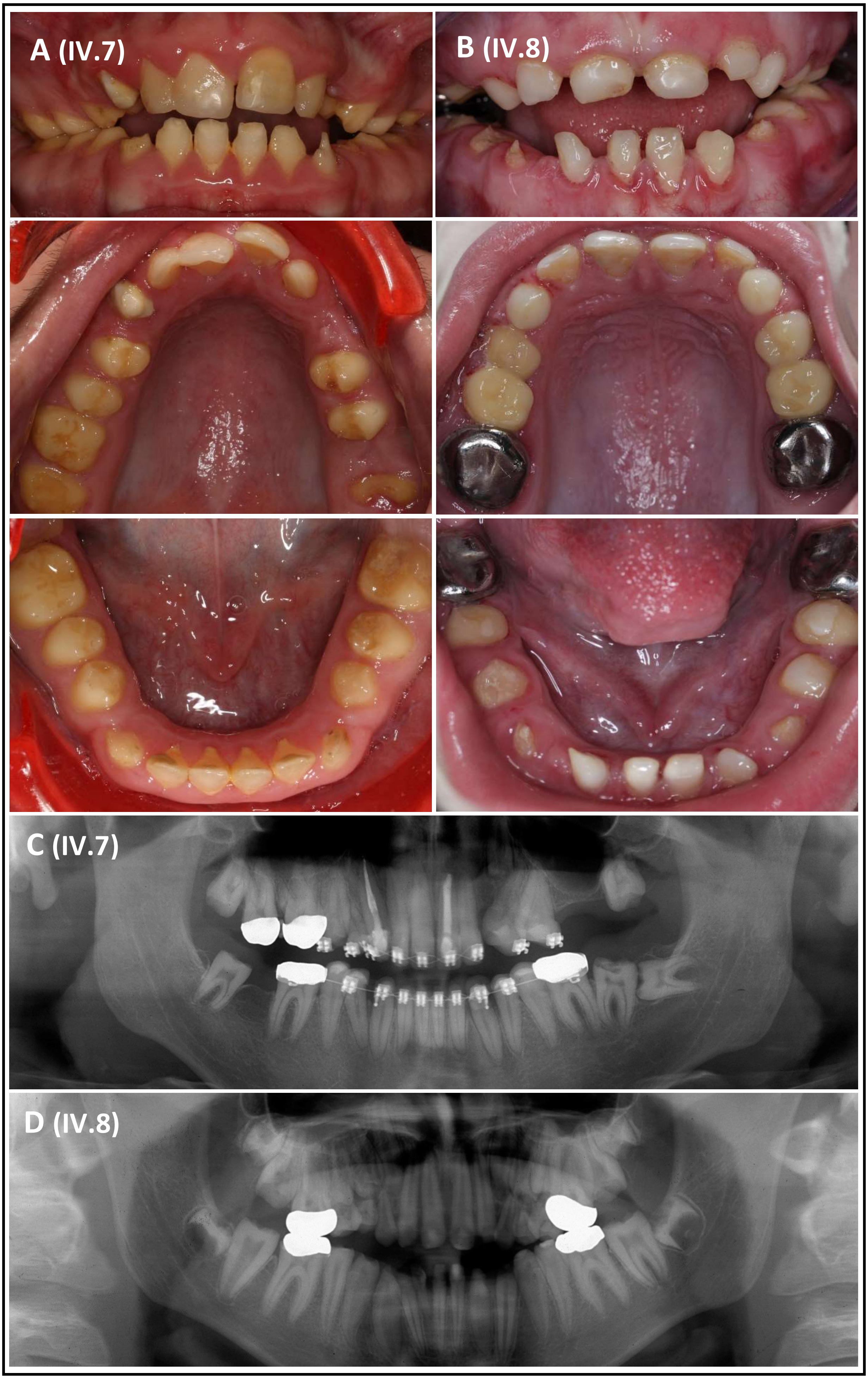

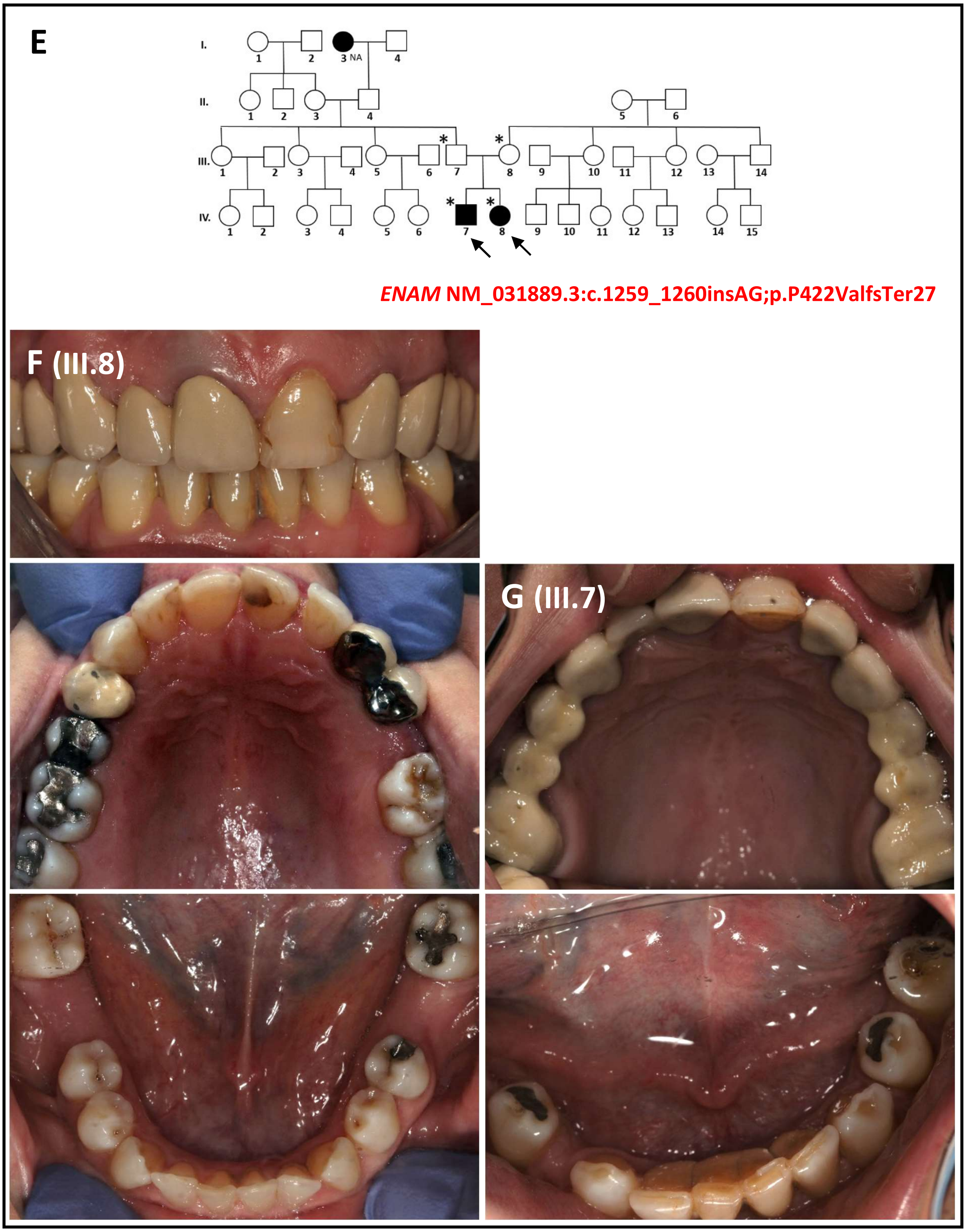

Figure S5. Family 5: **(A)** The 15-year-old boy (IV.7) and **(B)** his 11-year-and-4-month-old sister (IV.8) both exhibit severe, generalized enamel thinning, along with a pronounced anterior open bite. The upper anterior teeth of both patients are restored with composite resin. Additionally, the girl's permanent first molars are fitted with stainless steel crowns due to significant attrition. **(C, D)** In both panoramic radiographs, the enamel is poorly visualized. The boy's upper right permanent canine and upper left permanent central incisor have undergone endodontic treatment. **(E)** The family pedigree shows that both affected siblings (IV.7 and IV.8) are homozygous for the *ENAM* variant (NM_031889.3:c.1259_1260insAG;p.P422ValfsTer27). **(F)** Their mother (III.8) and **(G)** their father (III:7), both of whom have prosthetic crowns, were later confirmed to be heterozygous for the same disease-causing *ENAM* variant.

FAMILY 6

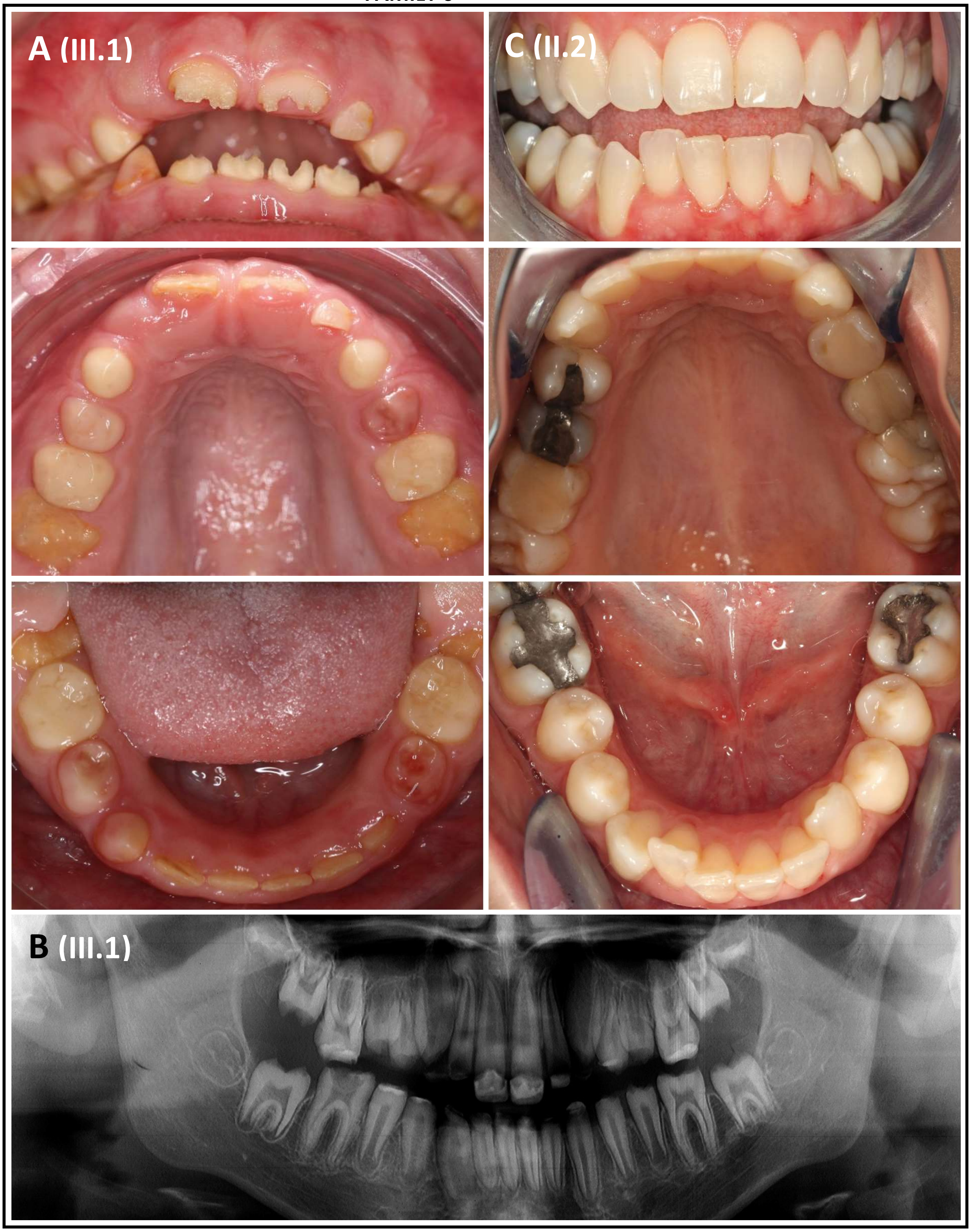

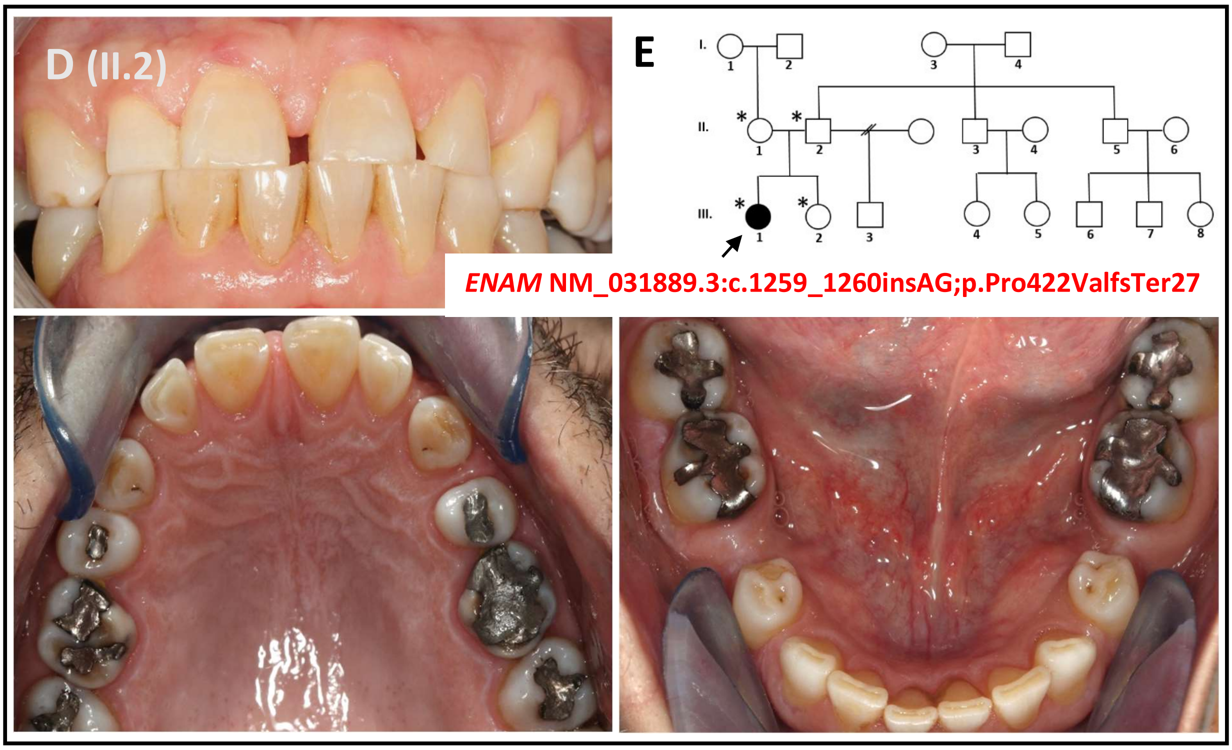

Figure S6. Family 6: **(A)** The 8-year-old girl (III.1) exhibits extensive hypoplasia of the mixed dentition along with an anterior open bite. The thinness of the enamel is evident on the newly erupted central incisors. **(B)** No enamel is visible on the panoramic radiograph taken at the age of 9. Neither **(C)** her mother (II.1) nor **(D)** her father (II.2) exhibits an aberrant enamel phenotype. **(E)** In the family pedigree, the girl (III.1) is found to be homozygous for the *ENAM* variant (NM_031889.3:c.1259_1260insAG;p.Pro422ValfsTer27). Both parents (II:1 and II:2), as well as her younger sister (III.2), who was still a baby at the time, were later found to be heterozygous for the same *ENAM* variant. The arrow indicates the proband, the asterisk (*) indicates participating family members, and "NA" denotes non-available family member.

FAMILY 7

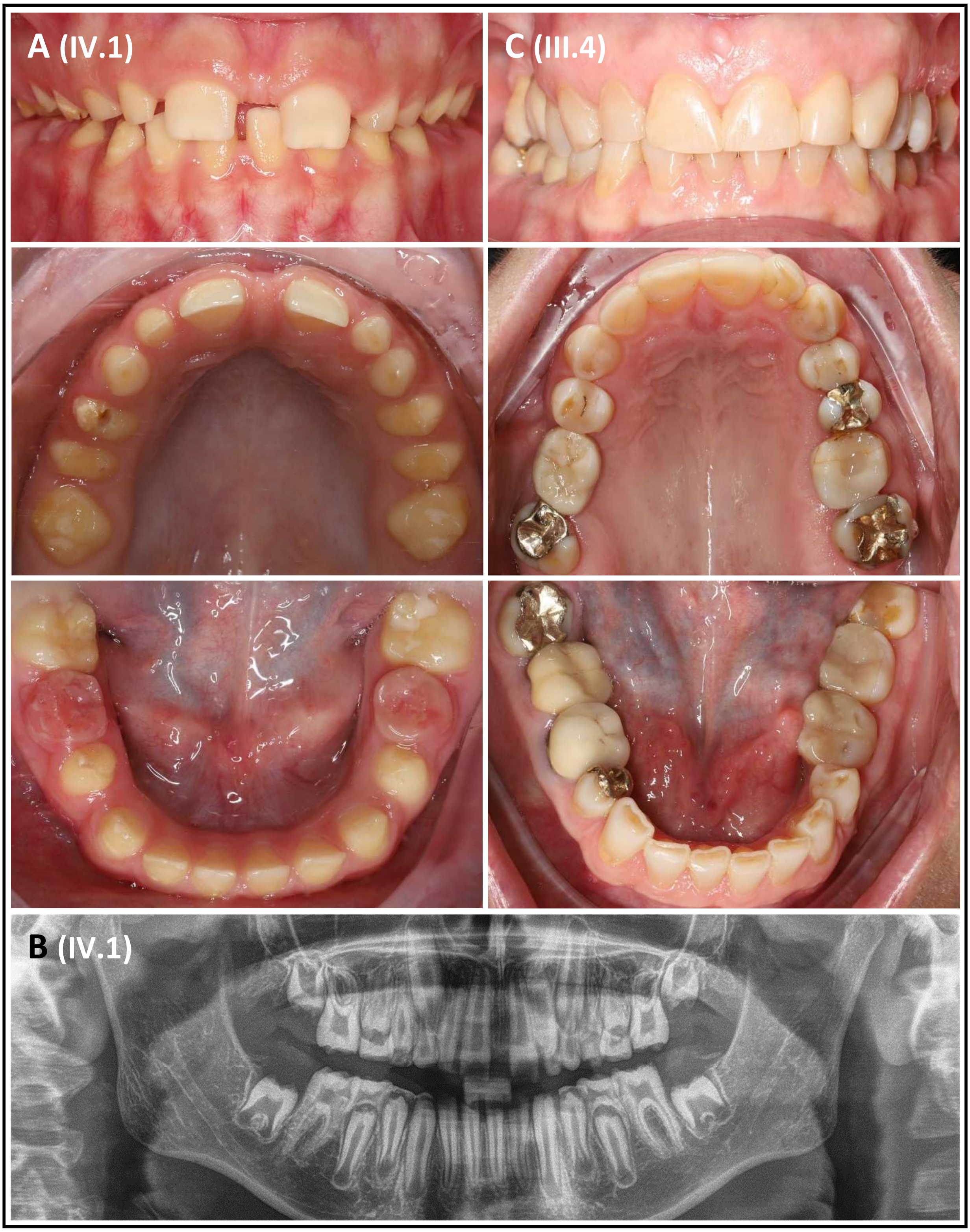

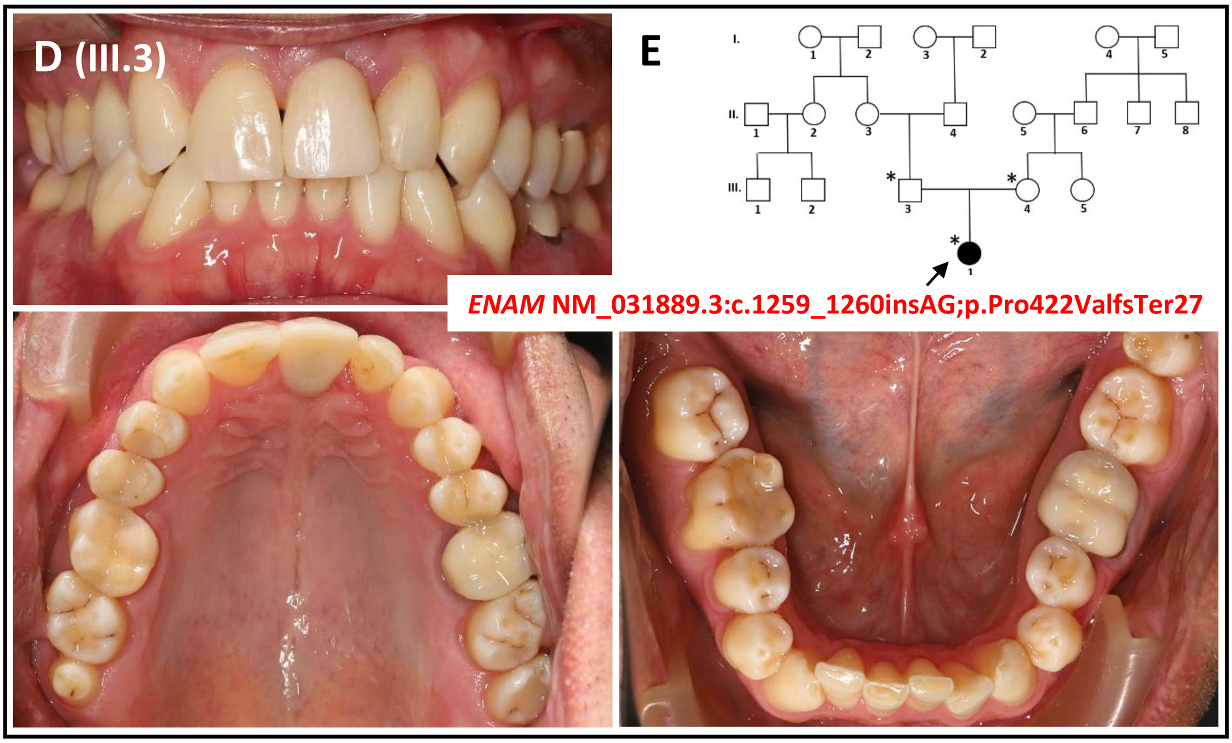

Figure S7. Family 7: **(A)** Clinical photographs of the 10-year-and-8-month-old girl (IV:1) depict severely hypoplastic enamel and interdental spacing. **(B)** A panoramic radiograph shows mixed dentition with little or no enamel, and noticeably enlarged pulp chambers. **(C)** The girl's mother's (III.4) and **(D)** father's teeth (III.3) do not exhibit developmentally aberrant enamel. **(E)** In the family pedigree, the girl (IV.1) is found to carry homozygous *ENAM* variant (NM_031889.3:c.1259_1260insAG;p.Pro422ValfsTer27). Both parents (III.3 and III.4) were later confirmed to be heterozygous for the same *ENAM* variant. The arrow indicates the proband, and the asterisk (*) indicates participating family members.

FAMILY 8

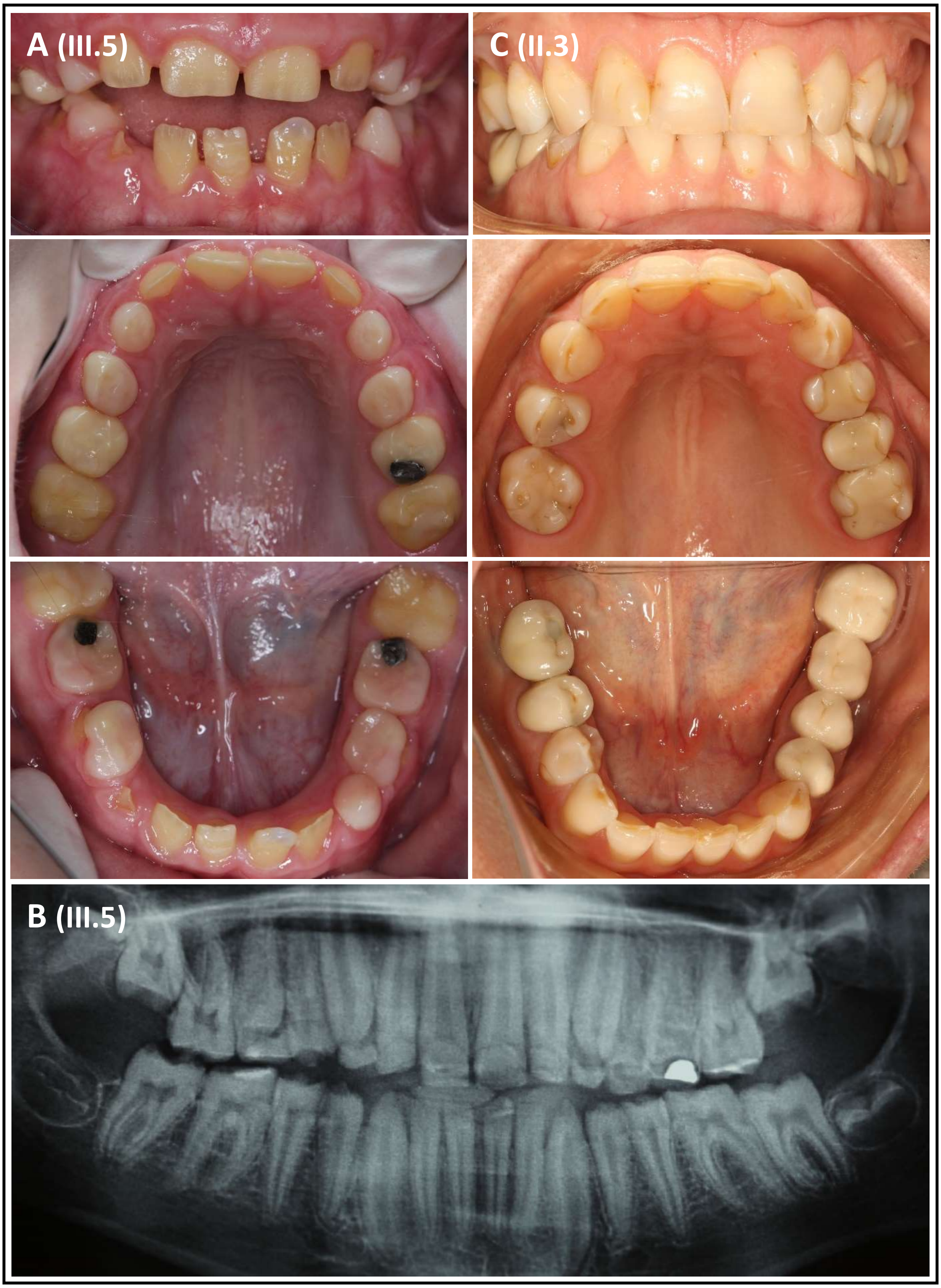

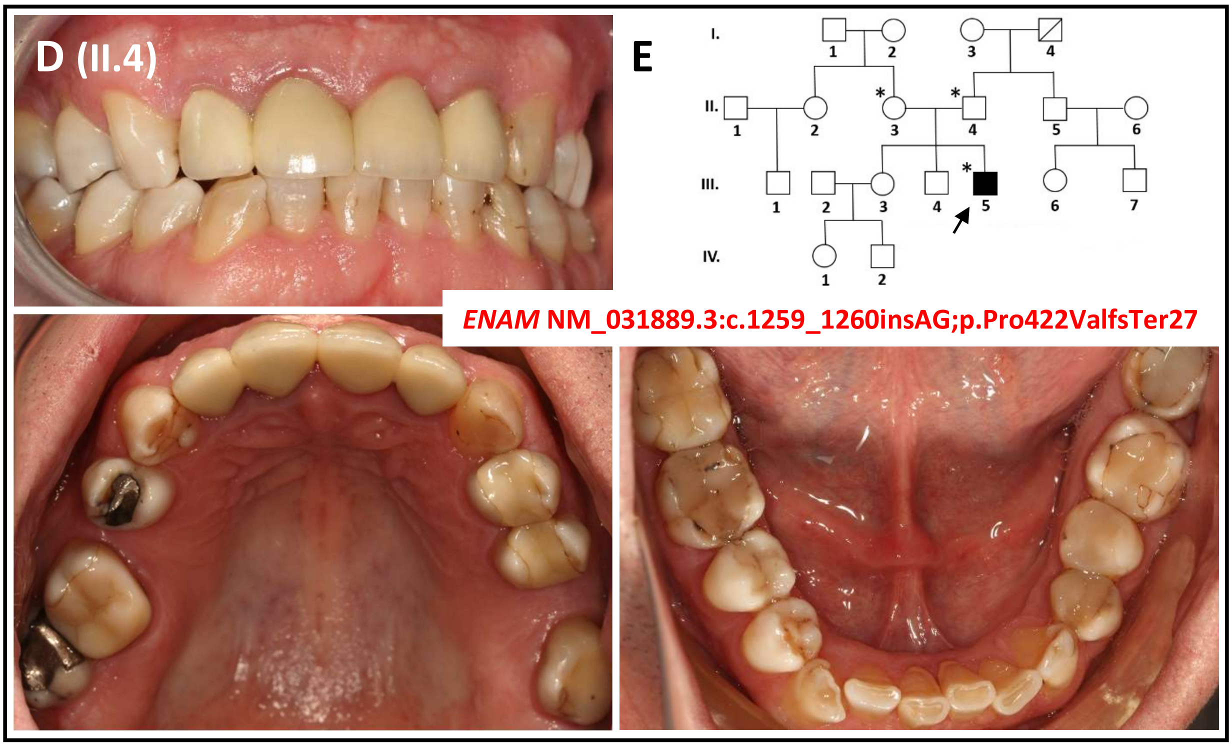

Figure S8. Family 8: **(A)** Clinical photographs of the 10-year-and-3-month-old boy (III:5) show generalized thin, hypoplastic enamel and an anterior open bite. **(B)** On the panoramic radiograph, taken at the age of 12 years, very little enamel is evident – either because it is absent or does not contrast with dentine. **(C)** His mother's (II:3) and **(D)** his father's teeth (II:4) either have extensive restorations or are covered with crowns or veneers. **(E)** In the family pedigree, the boy was found to be homozygous for *ENAM* variant (NM_031889.3:c.1259_1260insAG;p.Pro422ValfsTer27). Both parents were heterozygous for the same *ENAM* variant. The arrow indicates the proband, and the asterisk (*) indicates participating family members.

FAMILY 9

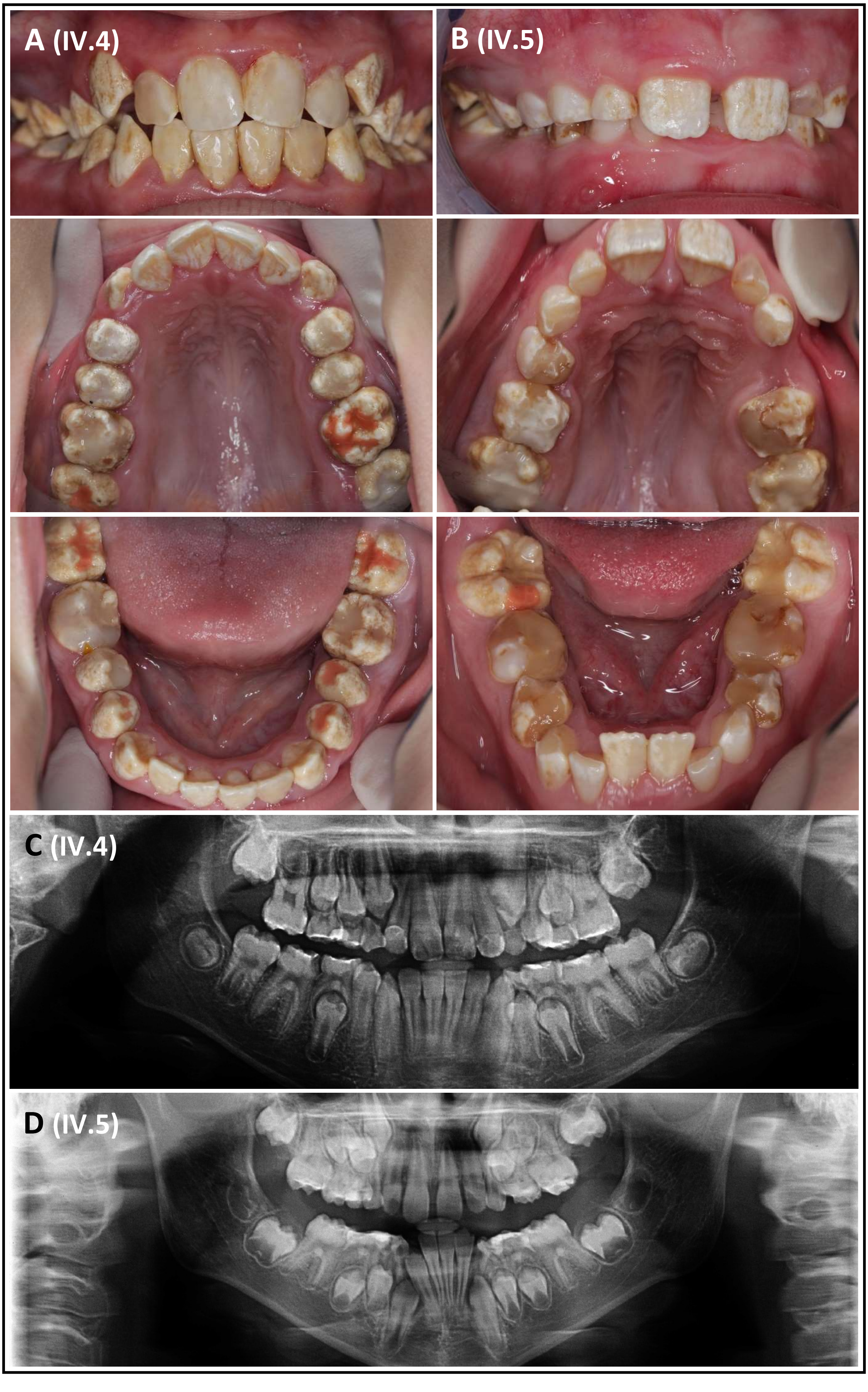

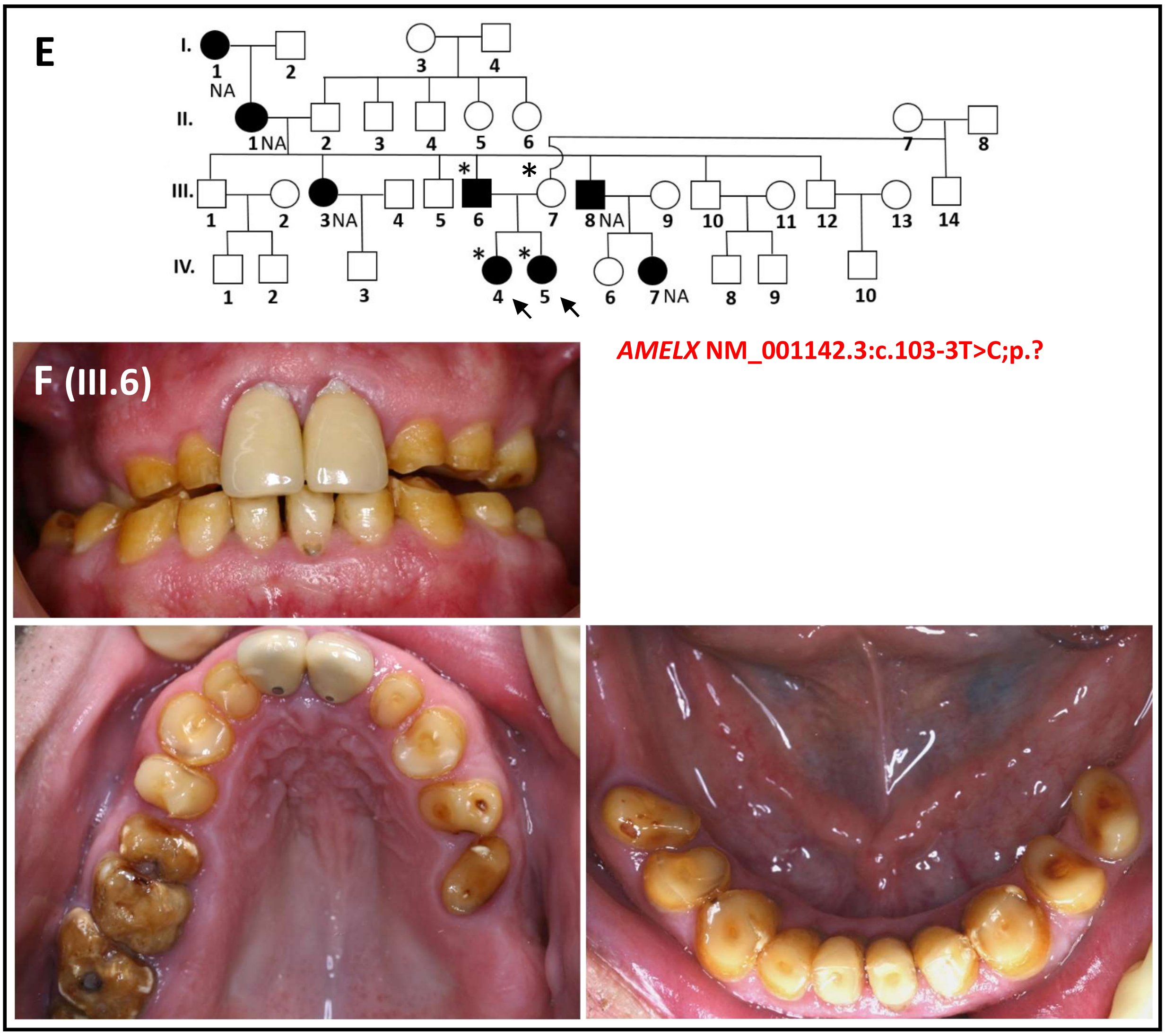

Figure S9. Family 9: **(A)** Clinical photographs of the 11-year-and-6-month-old girl (IV.4) and **(B)** her 6-year-and-9-month-old sister (IV.5) show similarly altered enamel, with a chalky-white to yellowish color featuring hypoplastic defects and marked hypomineralization. Both panoramic radiographs, **(C)** taken at the age of 9 years and **(D)** 6 years and 9 months, respectively, show enamel of adequate thickness but with radiopacity more similar to dentine. **(E)** The family pedigree indicates an X-linked mode of inheritance. Both sisters (IV.4 and IV.5) were identified as heterozygous for an *AMELX* variant (NM_182680.1:c.103-3T>C;p.?). **(F)** The probands' father (III.6), who was later found to be hemizygous for the same *AMELX* variant, shows teeth with developmentally aberrant enamel. The mother's teeth (III.7) developed normally. The arrows indicate probands, the asterisk (*) indicates participating family members, and "NA" denotes non-available family members.

FAMILY 10

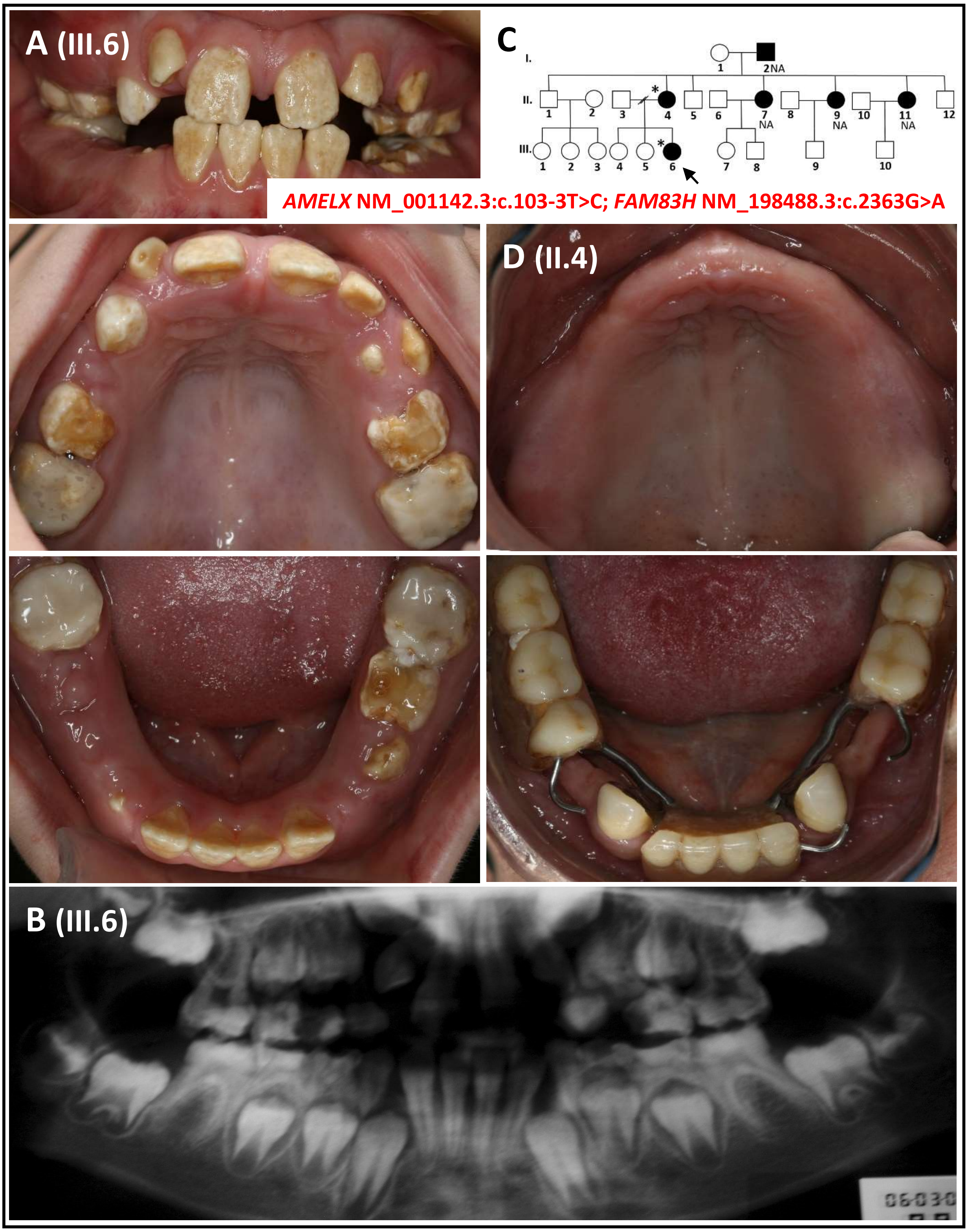

Figure S10. Family 10: **(A)** The hypomineralized mixed dentition of the 9-year-and-10-month-old girl (III.6) reveals chalky-white to yellowish hypoplastic enamel on all deciduous and permanent teeth. Profound attrition is observed on the occlusal surfaces of posterior teeth, with extensive glass ionomer fillings covering the permanent first molars. **(B)** The panoramic radiograph shows enamel of normal thickness but lacking contrast between enamel and dentine. **(C)** The family pedigree indicates an X-linked mode of inheritance: the girl (III.6) and her mother (II.4) are heterozygous for the *AMELX* variant (NM_182680.1:c.103-3T>C;p.?) and an additional *FAM83H* variant (NM_198488.3:c.2363G>A;p.Ser788Asn). **(D)** The proband’s mother (II.4) had no crowns available for inspection. Her upper jaw was toothless, and only canine teeth and a removable partial denture were present in the lower jaw.

FAMILY 11

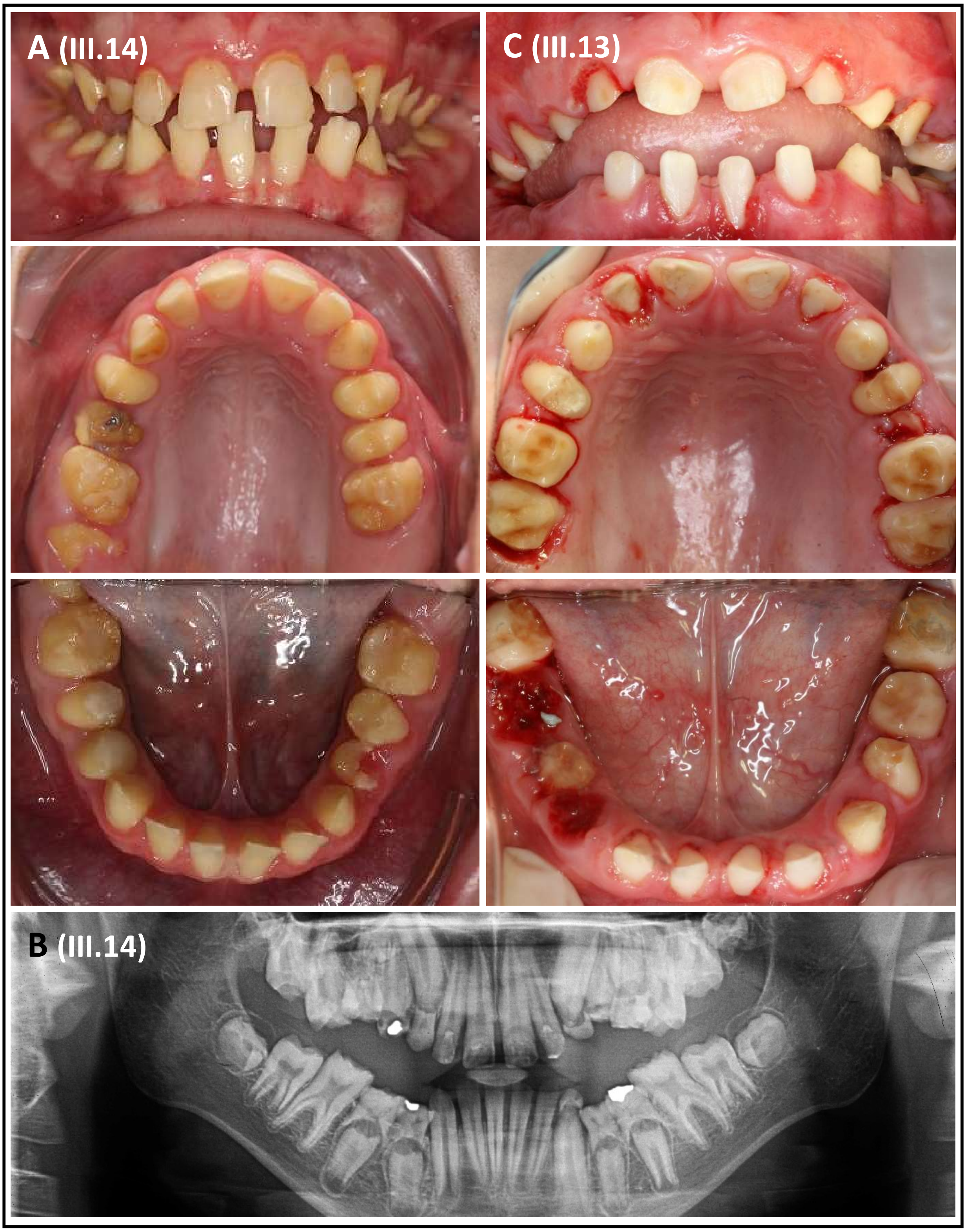

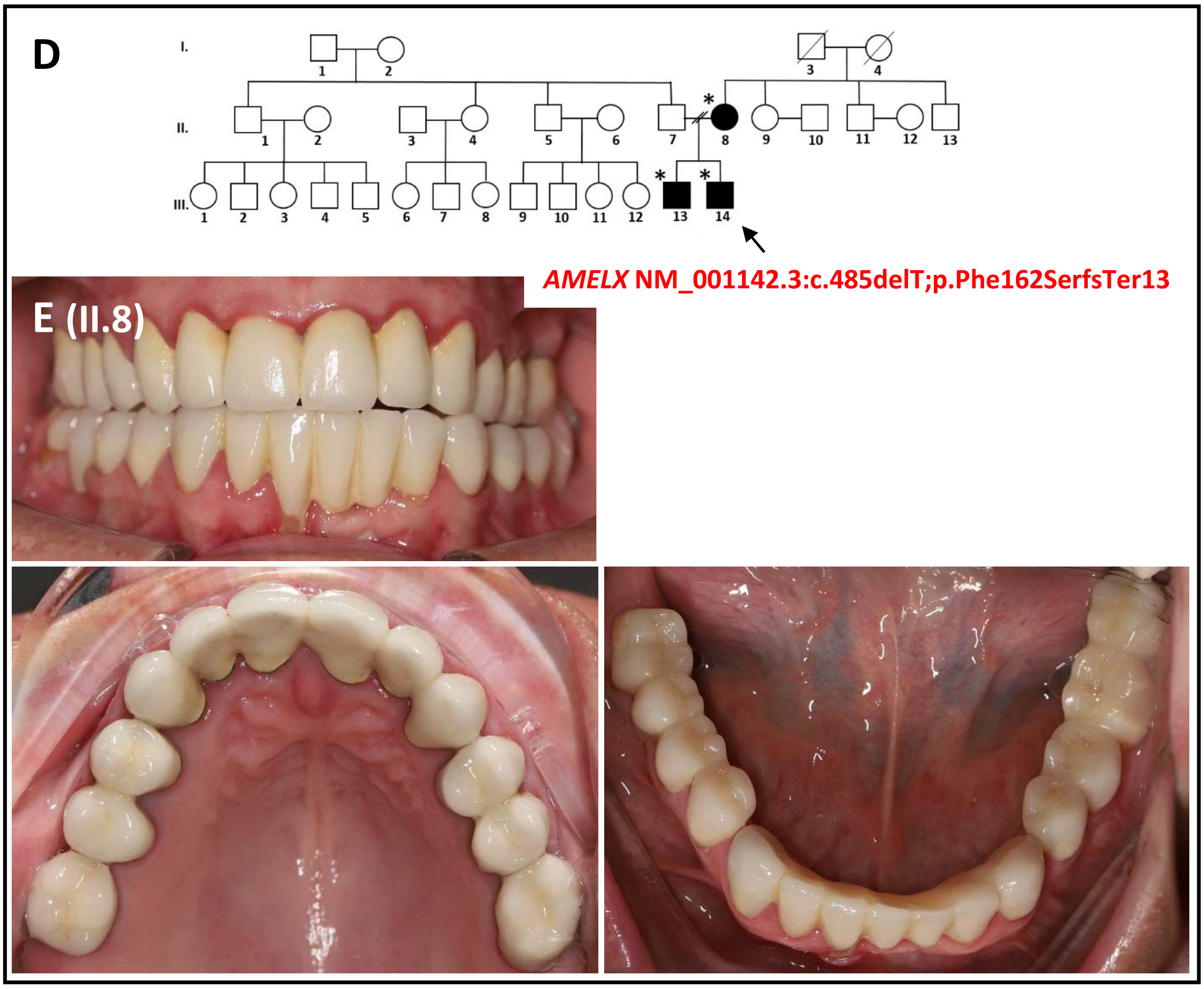

Figure S11. Family 11: **(A)** The permanent dentition of the 13-year-old boy (III:14) shows generalized thin, hypoplastic enamel with prominent interdental spacing. **(B)** Very little enamel is evident on the panoramic radiograph. The first permanent molars appear taurodontic. **(C)** The boy's brother's teeth (III.13) also show developmentally aberrant enamel, an open bite, and marginal gingivitis. **(D)** The family pedigree indicates an X-linked mode of inheritance: the boy (III.14) and his older brother (III.13) carry an *AMELX* variant (NM_001142.2:c.485delT;p.Phe162SerfsTer13) in hemizygous form, while their mother (II.8) carries the same *AMELX* variant in heterozygous form. **(E)** The boy's mother's teeth (II.8) lack crowns for inspection. A dental examination of the father (II.7) was not possible. The arrow indicates the proband, and the asterisk (*) denotes participating family members.

FAMILY 12

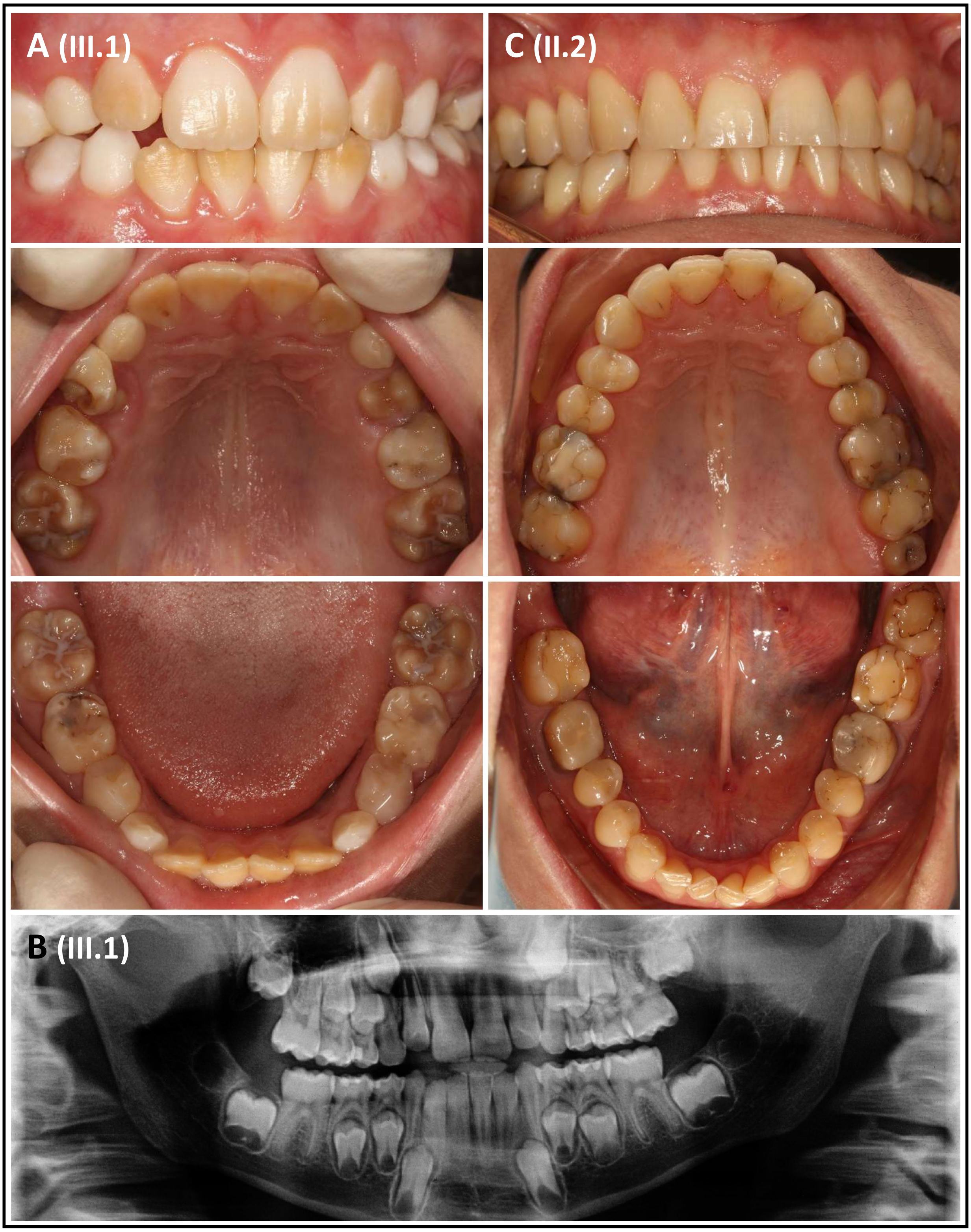

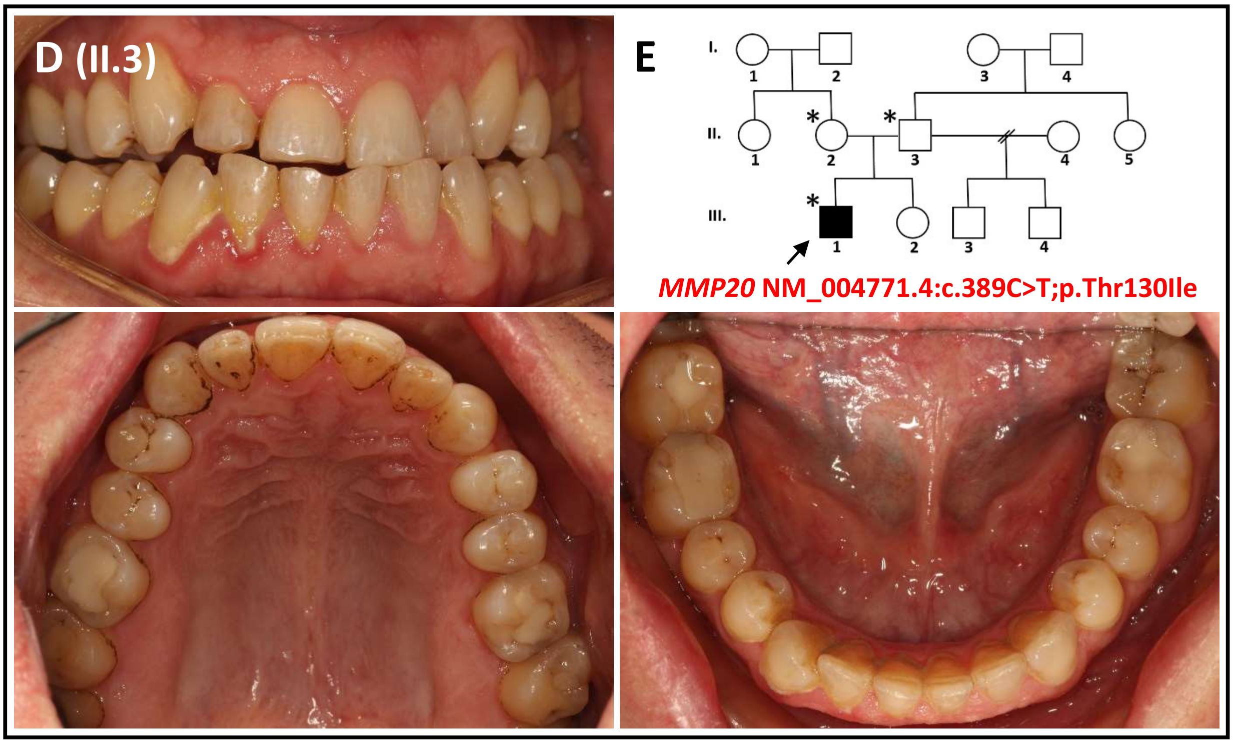

Figure S12. Family 12: **(A)** The mixed dentition of the 9-year-old boy (III.1) shows a generalized yellowish dull discoloration of the enamel, especially in the permanent teeth. **(B)** The panoramic radiograph reveals reduced contrast between enamel and dentine. In both parents, **(C)** his mother's teeth (II.2) and **(D)** his father's teeth (II.3), no aberrant enamel phenotype is detected. **(E)** The family pedigree shows that the boy (III.1) carries a homozygous *MMP20* variant (NM_004771.4:c.389C>T;p.Thr130Ile). Both parents (II.2 and II.3) were later found to be heterozygous for the same *MMP20* variant. The arrow indicates the proband, and the asterisk (*) denotes participating family members.

FAMILY 13

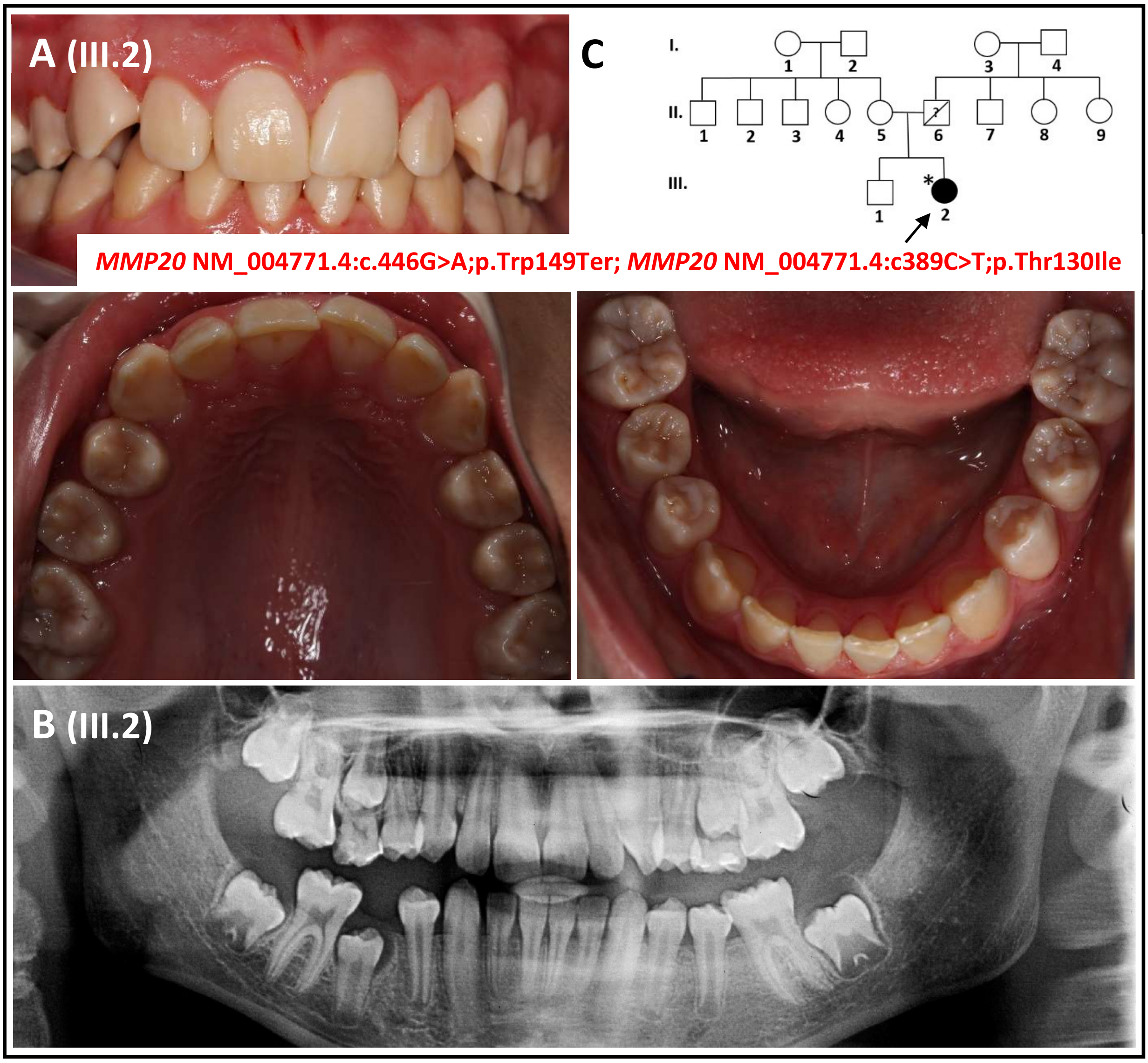

Figure S13. Family 13: **(A)** The enamel of the 13-year-old girl (III.2) shows an opaque and creamy appearance. **(B)** A panoramic radiograph taken at the age of 12 years reveals normal morphology of tooth germs, with limited contrast between enamel and dentine. **(C)** The family pedigree shows that the patient carries compound heterozygous *MMP20* variants [NM_004771.4:c.446G>A;p.Trp149Ter and NM_004771.4:c.389C>T;p.Thr130Ile]. Her father (II.6) passed away, and DNA from her unaffected mother (II.5) was not available. The arrow indicates the proband, and asterisk (*) denotes participating family member.
